# An M0 macrophage-related prognostic model for hepatocellular carcinoma

**DOI:** 10.1186/s12885-022-09872-y

**Published:** 2022-07-19

**Authors:** Yiya Zhang, Ju Zou, Ruochan Chen

**Affiliations:** 1 Hunan Key Laboratory of Viral Hepatitis, Xiangya Hospital, Central South University, Changsha, 410008 Hunan China; 2 Department of Dermatology, Xiangya Hospital, Central South University, Changsha, 410008 Hunan China; 3 Hunan Key Laboratory of Aging Biology, Xiangya Hospital, Central South University, Changsha, 410008 Hunan China; 4 Department of Infectious Disease, Xiangya Hospital, Central South University, Changsha, 410008 Hunan China

**Keywords:** Macrophage, M0 macrophage-related gene, Risk score, Hepatocellular carcinoma, Therapy, Prognosis

## Abstract

**Background:**

The role of M0 macrophages and their related genes in the prognosis of hepatocellular carcinoma (HCC) remains poorly characterized.

**Methods:**

Multidimensional bioinformatic methods were used to construct a risk score model using M0 macrophage-related genes (M0RGs).

**Results:**

Infiltration of M0 macrophages was significantly higher in HCC tissues than in normal liver tissues (*P* = 2.299e-07). Further analysis revealed 35 M0RGs that were associated with HCC prognosis; two M0RGs (*OLA1* and *ATIC*) were constructed and validated as a prognostic signature for overall survival of patients with HCC. Survival analysis revealed the positive relationship between the M0RG signature and unfavorable prognosis. Correlation analysis showed that this risk model had positive associations with clinicopathological characteristics, somatic gene mutations, immune cell infiltration, immune checkpoint inhibitor targets, and efficacy of common drugs.

**Conclusions:**

The constructed M0RG-based risk model may be promising for the clinical prediction of prognoses and therapeutic responses in patients with HCC.

**Supplementary Information:**

The online version contains supplementary material available at 10.1186/s12885-022-09872-y.

## Introduction

Hepatocellular carcinoma (HCC) ranks sixth in terms of incidence among all types of tumors worldwide and has a high mortality rate [1]. The 5-year survival rate of patients is only 5–7%, and the recurrence rate of HCC is up to 60–70% [2]. HCC tumorigenesis is driven by intrinsic factors, such as mutations in liver parenchymal cells, and external factors, including interactions between tumor cells and surrounding stromal cells, immune cells, and noncellular components [3]. Tumor cells and adjacent immune cells, stromal cells, and the extracellular matrix constitute a complex and dynamic network of the tumor immune microenvironment (TIME). The components of the TIME interact to produce growth factors, cytokines, and chemokines that participate in immunosuppression, thereby promoting the development, recurrence, and metastasis of HCC cells [4, 5].

Various immune cells in the TIME, such as tumor-associated macrophages (TAMs), tumor-associated neutrophils, tumor-infiltrating lymphocytes, regulatory T cells (Tregs), CD8^+^ cytotoxic T lymphocytes, and natural killer cells, are active players in HCC pathogenesis. TAMs, as a critical factor of tumor-related inflammation, can be polarized into disparate functional phenotypes, among which M1 macrophages, which are induced by interferon alone or with lipopolysaccharide, and M2 macrophages, which are induced by IL-4 and IL-13, are the most studied subgroups. Classically activated macrophages with the M1 phenotype can stimulate antitumor immune responses by presenting antigens to adaptive immune cells, producing proinflammatory cytokines, and phagocytosing tumor cells [6–10]. TAMs polarized into the M2 phenotype can promote HCC progression by upregulating cytokine secretion and protein expression. Resting-state macrophages (M0), derived from the bone marrow, are usually considered precursors of polarized macrophages. The prevailing view is that both M1 and M2 macrophages are generated from M0, and M0 is only a resting state of macrophages, without a specific function before their polarization. However, a recent study on immunophenotyping of glioma-associated macrophages versus matched blood monocytes, health donor monocytes, normal brain microglia, nonpolarized M0 macrophages, and polarized M1 and M2 macrophages has indicated that macrophages that infiltrate into glioma tissues maintain a continuum state between the M1- and M2-like phenotypes and resemble M0 macrophages [11]. Further analysis of glioma data from The Cancer Genome Atlas (TCGA) and the Chinese Glioma Genome Atlas databases confirmed that differentiation of M0-like macrophages, rather than M1 or M2 macrophages, is associated with a high-grade tumor and a poor prognosis in glioma [12]. These studies indicated the tumorigenic role of M0 macrophages.

However, cellular infiltration and molecular features of M0 macrophages and their association with clinicopathological characteristics of HCC have not been explored. Bioinformatics tools can facilitate the efficient prediction of the composition of and changes in the TIME [13]. Therefore, in this study, we used bioinformatic tools to explore the clinical significance of M0 macrophages, association between the TIME and tumorigenesis, and the effects of immunotherapy and chemotherapy on HCC [14, 15]. This study may help advance our understanding of the role of M0 macrophages in HCC, and the constructed risk model may be promising for clinical prediction of the prognosis and therapeutic efficacy in patients with HCC.

## Materials and methods

### Data acquisition

The gene expression profiles and clinical parameters of patients with HCC were obtained from TCGA, International Cancer Genome Consortium (ICGC) and GSE datasets. Somatic mutation and copy number variation (CNV) profiles were obtained from TCGA data portal (https://portal.gdc.cancer.gov/). Somatic mutation data were analyzed using “maftools” in the R package. Significant amplifications or deletions of the copy number variant were detected using GISTIC 2.0 with a false discovery rate threshold of < 0.05. As the study used only publicly available data from TCGA, there was no requirement for an ethical approval.

### Analysis of infiltrating immune cells in HCC

Data on infiltrating immune cells in HCC were obtained using CIBERSORT. Differences in levels of infiltrating immune cells between high- and low-risk HCC samples were examined using the Wilcoxon test. The expression of M0-related genes (M0RGs) was calculated using Pearson’s correlation analysis with |*R*|> 0.3 and *P* < 0.05. Gene ontology (GO) enrichment analysis was used to reveal the M0RGs-related biological functions in HCC.

### Establishment of M0RG signatures

Cox analysis and LASSO regression analysis were performed to establish M0RG signatures in TCGA dataset, and then, the results were verified in the ICGC dataset. The risk score was calculated using M0RG expression and coefficient values as follows: coefficient 1 × M0RG 1 expression + coefficient 2 × M0RG 2 expression + coefficient 3 × M0RG 3 expression.

The best cutoff value derived from the receiver operating characteristic (ROC) curve was used to divide the patients with HCC into low-risk and high-risk groups (Figure S3). For survival analysis, Kaplan–Meier survival curves were constructed for both the low- and high-risk groups in both the cohorts using the R package “survival.” A two-sided log-rank test was used with *P* < 0.05 considered significant. The prognostic value of the M0RG signatures was examined using “survival.” Using the R package “survivalROC,” a survival ROC curve was constructed to verify the prognostic performance.

A nomogram was constructed using the risk score and other clinical parameters for each cohort. ROC curves were used to compare the prognostic value of risk scores with that of other clinical features using the “ROC” package in the R software 4.0.5.

### Gene set enrichment analysis (GSEA)

Enrichment terms were analyzed in the entire TCGA cohort using the GSEA software version 4.1.0 (http://www.gsea-msigdb.org/gsea/index.jsp, Cambridge, MA, USA) to reveal M0RG-related pathways. The gene sets of “c2.cp.kegg.v7.4.symbols.gmt” were selected for GSEA. Significance was indicated by *P* < 0.05 and a false discovery rate of < 0.05.

### Expression of risk M0RGs in immune cells

tSNE analysis was performed using web tools (http://hcc.cancer-pku.cn/) to examine the expression of risk genes in immune cells in HCC.

### Efficacy analysis of immune checkpoint inhibitors (ICIs) in HCC

The correlations between known ICI targets (TIM-3, IDO1, CTLA4, PD-1, PD-L1, and PD-L2) and our signature were analyzed to explore the possible roles of M0RGs and the risk signature in ICI efficacy in HCC.

### Evaluation of potential model significance in clinical treatment

To evaluate the potential significance of the model in the clinical treatment of HCC, we calculated the half-maximal inhibitory concentrations (IC_50_s) of commonly used chemotherapeutic drugs (etoposide, A.443654, doxorubicin, gemcitabine, cisplatin, dasatinib, gefitinib, metformin, and rapamycin) using TCGA- liver hepatocellular carcinoma (LIHC) project dataset. The differences in the IC_50_ values between the high- and low-risk groups were evaluated using the Wilcoxon signed-rank test, and the results are shown as box drawings obtained using the “pRRophetic” and “ggplot2” tools in the R software.

### Statistical analysis

The Wilcoxon signed-rank test was used for analysis of correlation between M0RGs and clinical characteristics of patients with HCC. The correlations among M0RGs, immune cells, and ICIs were analyzed using Spearman’s correlation coefficient. Kaplan–Meier curves were used for survival analysis.

## Results

### M0RGs in HCC

First, infiltration of M0 macrophages was analyzed in HCC using TCGA dataset. As shown in Figure S1A, infiltration of M0 macrophages was significantly higher in HCC tissues than in normal liver tissues. The patients with HCC with high infiltration of M0 macrophages showed a poor overall survival (OS) (Figure S1B). Next, the relationships between infiltration of M0 macrophages and clinical characteristics of HCC were analyzed. The results showed that infiltration of M0 macrophages was associated with the survival status, stage, and T stage (Figure S1C).

**Fig. 1:**
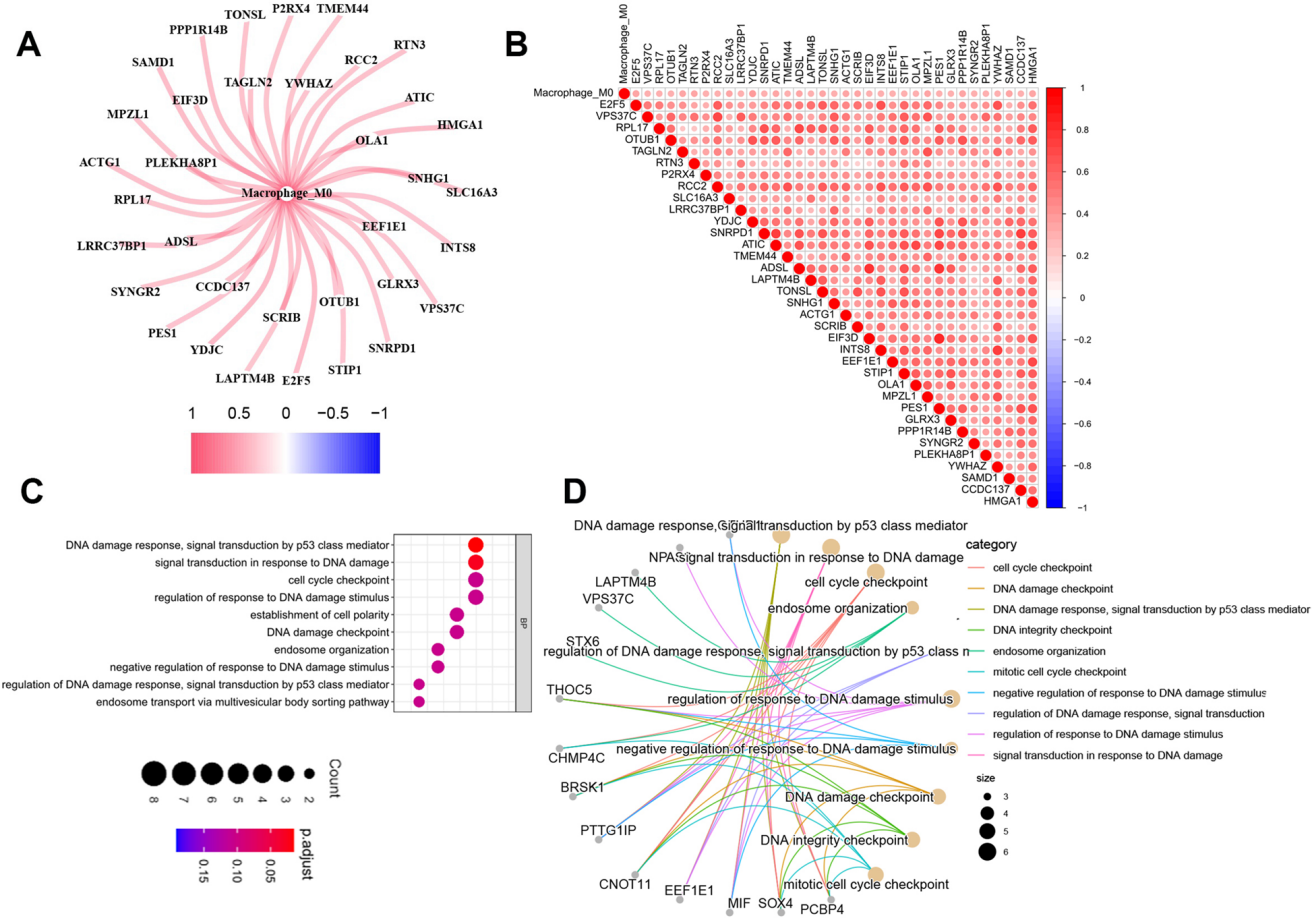
M0 macrophages related genes in HCC. **A** and **B**, A correlation network involving the 35 prognosis-related M0RGs and M0 macrophages in the TCGA cohort. **C** and **D**, GO analyzed of the 35 M0RGs. M0RGs: M0 macrophages-related genes; TCGA: The Cancer Genome Atlas; Go: Gene Ontology.

Subsequently, we identified 99 M0RGs using Pearson’s analysis (Table S1), of which 35 M0RGs were associated with the prognosis of patients with HCC in both TCGA and ICGC datasets (Tables S2 and S3). The correlation network involving the 35 M0RGs and M0 macrophages in TCGA cohort is shown in Fig. 1A and B. GO analysis showed that the 35 M0RGs were enriched in DNA damage and cell cycle-related signaling pathways (Fig. 1C and D). Figure S2 shows the CNVs and mutation statuses of the 35 M0RGs.

### Establishment and validation of a M0RG prognostic signature for OS of patients with HCC

**Fig. 2:**
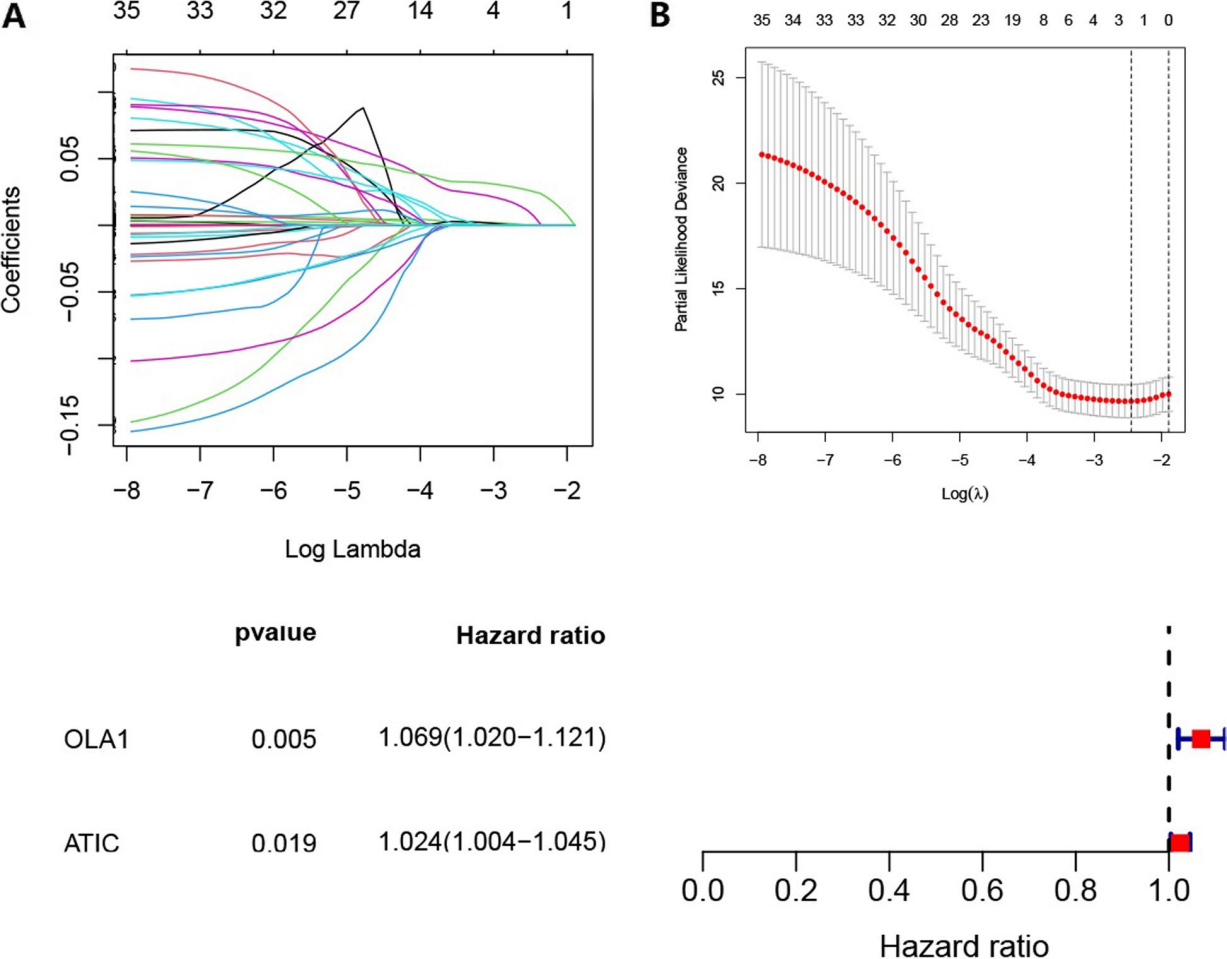
The M0RGs prognostic signature. **A**, Cross-validation for tuning parameter (lambda, screening in the LASSO regression model. **B**, LASSO coefficient profiles of 35 prognostic M0RGs. **C**, Forest plot of the seven DNA replication-related genes. M0RGs: M0 macrophages-related genes

**Fig. 3:**
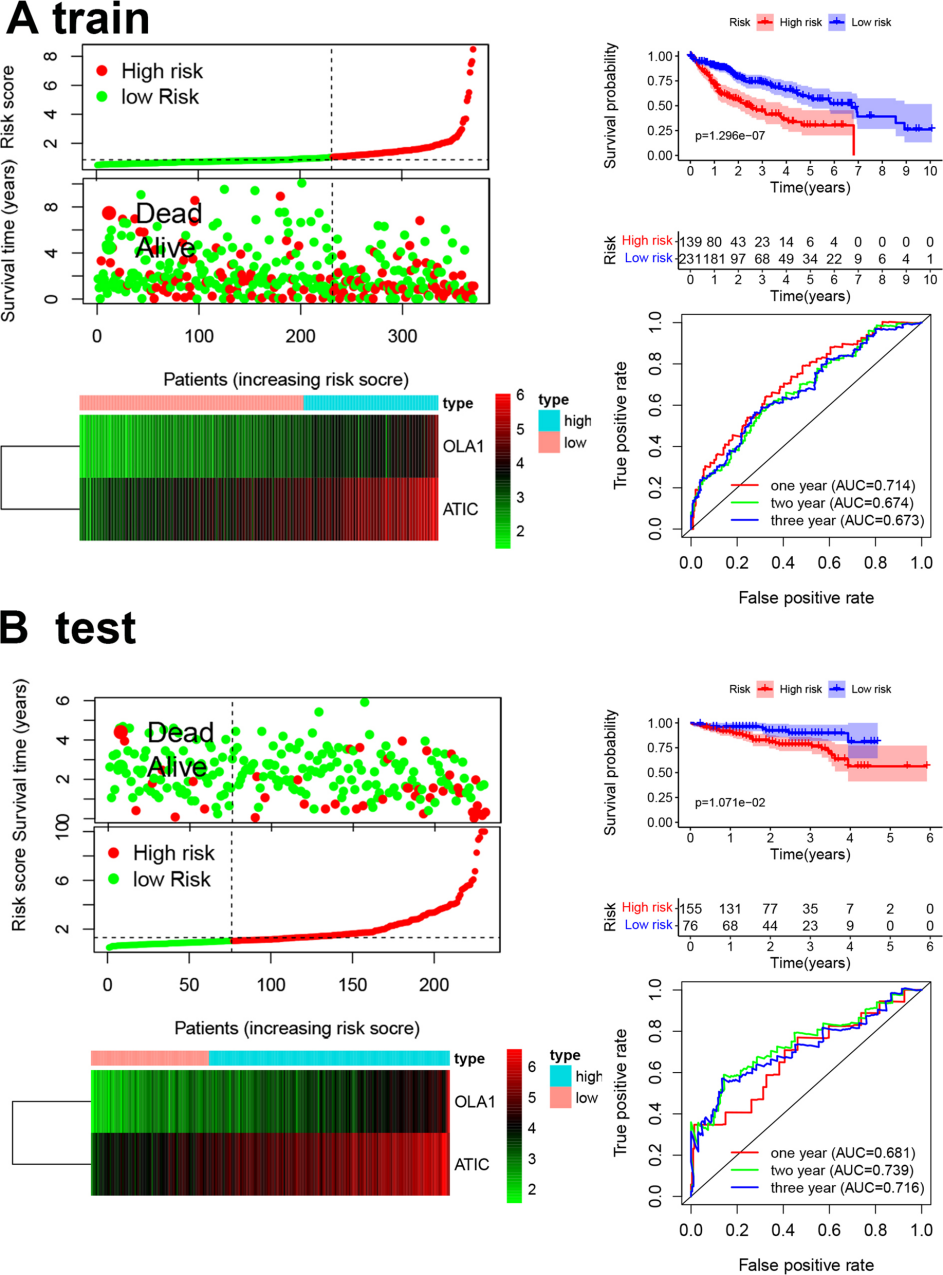
Prognostic model of the train (TCGA) cohort and test (ICGC) cohort. **A** Train set (**B**) Test set. Risk score of the high and low groups. Heatmap of the expression of 2 M0RGs. Survival analysis of the high and low groups. The AUC of the ROC. TCGA: The Cancer Genome Atlas; ICGC: International Cancer Genome Consortium; M0RGs: M0 macrophages-related genes; AUC: Area under curve; ROC: Receiver operating characteristic curve

The LASSO Cox algorithm was used to identify the most robust prognostic genes among the 35 candidate genes (Fig. 2A), and multivariate Cox regression analysis was performed to build prognostic signatures based on two M0RGs, Obg-like ATPase 1 (*OLA1*) and 5-aminoimidazole-4-carboxamide ribonucleotide formyl transferase/inosine monophosphate cyclohydrolase (*ATIC*) (Fig. 2B). The risk score was calculated as follows: risk score = *OLA1* × 0.0671 + *ATIC* × 0.0241. Next, the best cutoff value of the ROC curve was adopted to distinguish between the high- and low-risk groups (Figure S3). Survival analysis showed striking differences between the two groups in both the training TCGA and test ICGC datasets (Fig. 3A and B). The cut-off points of optimal separation of overall survival (OS) were also analyzed using the X-Tile software (Yale School of Medicine, CT, USA) (Figures S5 and S6) [16]. The mRNA expression of the two M0RGs in each sample is shown in Fig. 3A and B. The accuracy was evaluated based on the area under the curve (AUC) of the ROC curve, with AUC values of 0.714 at 1 year, 0.674 at 2 years, and 0.673 at 3 years in TCGA dataset and 0.681 at 1 year, 0.739 at 2 years, and 0.716 at 3 years in the ICGC dataset. We also validated the risk score in GSE14520 (Figure S4).

### Association between clinicopathological characteristics and the prognostic risk score

**Fig. 4:**
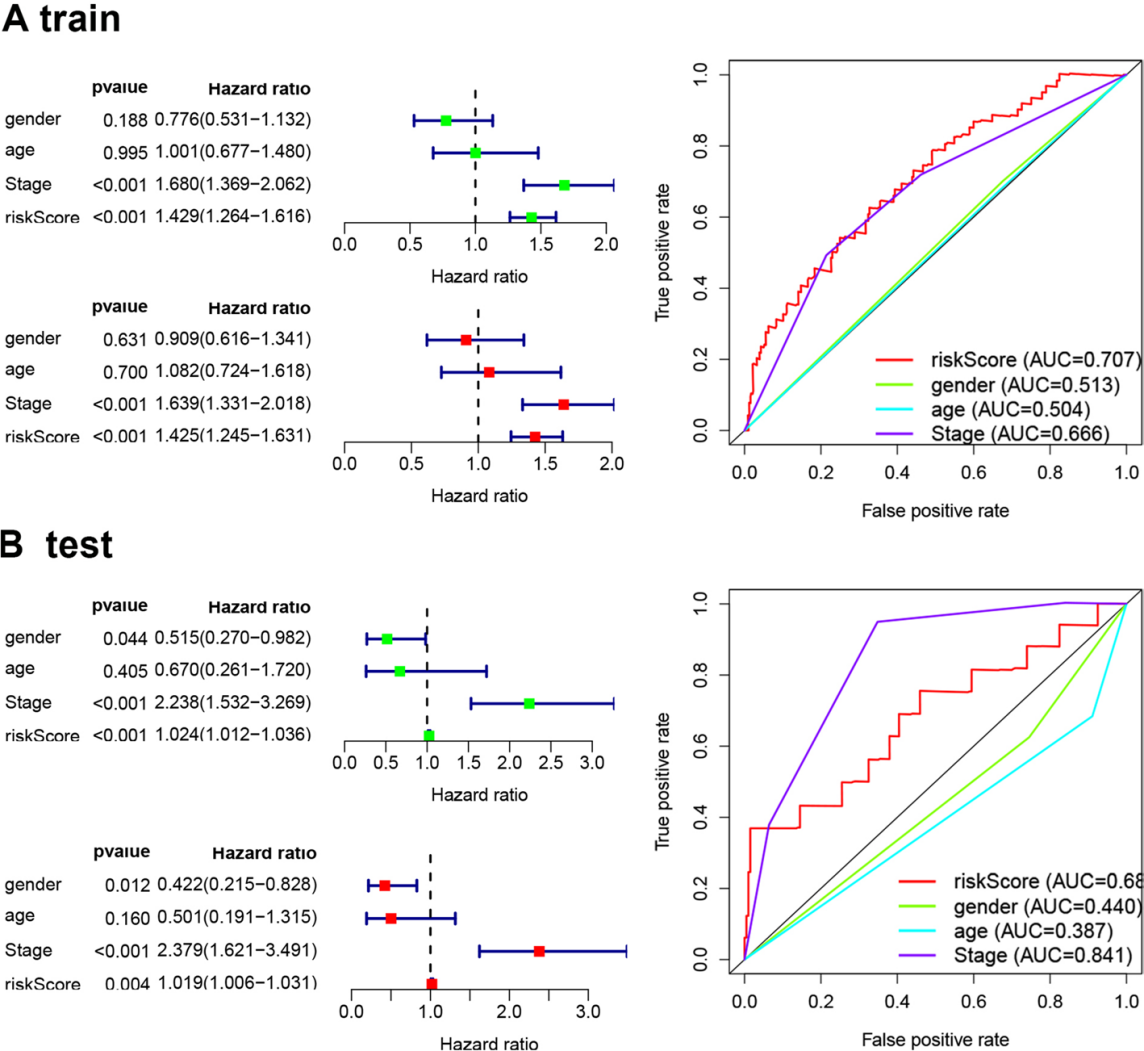
Association between the clinicopathological characteristics and prognostic risk score. **A**, Univariate and multivariate Cox regression analyses and ROC value in training group. **B**, Univariate and multivariate Cox regression analyses and ROC value in testing group. ROC: Receiver operating characteristic curve

To further verify the prognostic value of the risk signature, we explored the correlations between clinicopathological characteristics of patients with HCC and the risk signature. The univariate Cox regression analysis showed that the risk score and stage were significantly correlated with OS in the training set. Multivariate Cox regression analysis revealed that the risk score and stage were independent factors of HCC prognosis (Fig. 4A). Moreover, the AUC value for the risk Score was much higher than that for the other clinical characteristics (Fig. 4A). These results were also confirmed in the test set (Fig. 4B) and indicated that the risk model established based on the two M0RGs could be used as an independent prognostic factor for patients with HCC.

**Fig. 5:**
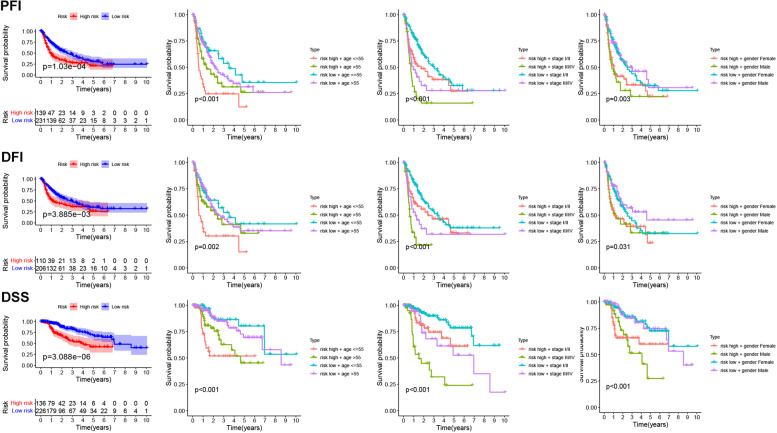
The prognosis of HCC patients with high/low risk score. HCC: Hepatocellular carcinoma

Furthermore, patients with HCC in the high-risk group showed a poor prognosis in terms of progression-free interval (PFI), disease-free interval (DFI), and disease-associated survival (DSS) (Fig. 5). Patients with HCC with a high risk also showed a poor prognosis in terms of the OS, PFI, DFI, and DSS for the male, female, age > 55 years, and age ≤ 55 years groups (Figs. 5 and S4).

### Construction and validation of a nomogram

**Fig. 6:**
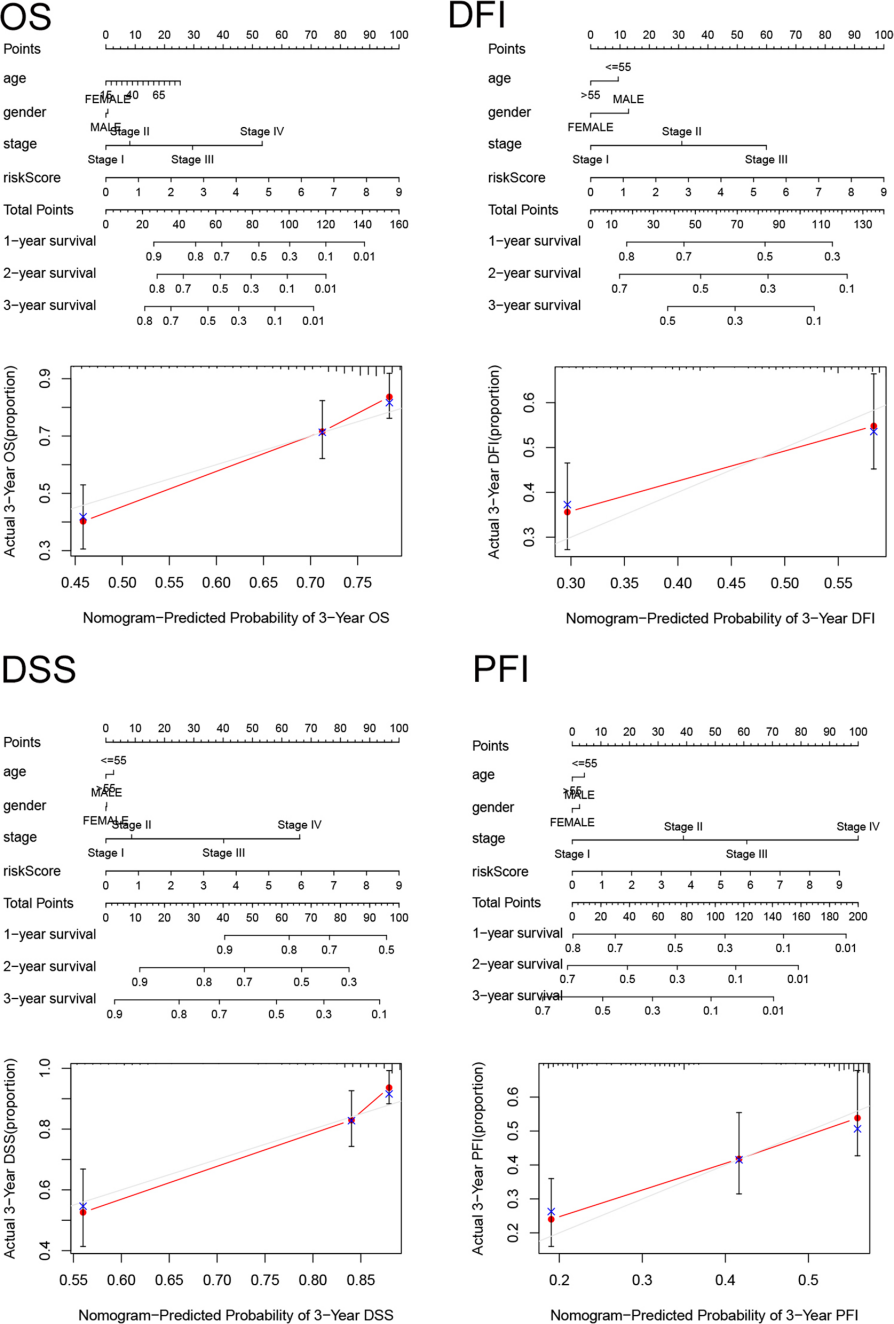
Construction of the nomogram in the TCGA dataset. The nomogram to predict the 1-, 2- and 3-year survival risk of HCC patients. The calibration curve of the 3-year survival

A nomogram associated with the OS of patients with HCC was established using TCGA dataset (Fig. 6) and externally validated in the ICGC dataset (Figure S9). The calibration curve indicated a high reliability of the nomogram (Fig. 6 and Figure S9). Similar results were obtained for the DFI, PFI, and DSS of patients with HCC. These results suggested that the prognostic model might be a good predictor of survival of patients with HCC. The C-index for discrimination was calculated in TCGA and ICGC (Figure S7).

### Relationship between M0RGs and immune cell infiltration

**Fig. 7:**
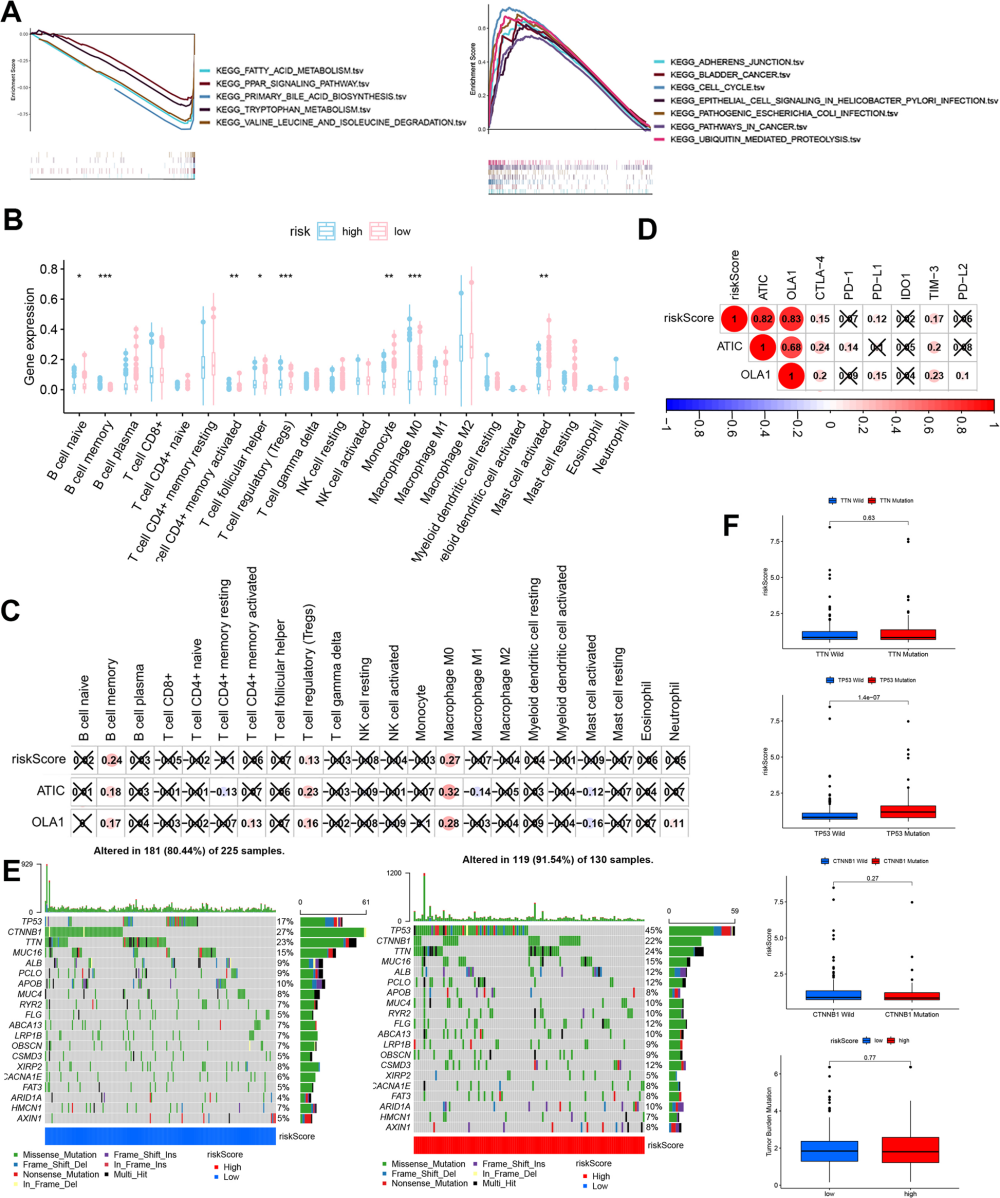
The Relationship between M0RGs and Immune infiltration, mutation state. **A**, GSEA analysis. **B**, immune cell in high/low groups. C, The relationship between immune cell and risk score. **D**, The relationship between risk score and ICB. **E**, The mutation in high/low risk group. **F**, The relationship between risk score and TP53, TTN, CTNNB1 mutation, TMB

GSEA results showed that cancer- and immune-related signaling pathways were enriched in the high-risk group (Fig. 7A). To further understand the association between the risk signature and immune cell infiltration, CIBERSORT analysis was conducted. The different infiltration of immune cells was observed in the high- and low-risk group (Fig. 7B). Moreover, B memory cells, Tregs, and M0 macrophages were positively associated with the risk score, according to Pearson’s analysis (Fig. 7C).

Next, we investigated the role of the M0RGs signature in predicting ICI therapeutic efficacy in HCC by evaluating the relationship between six well-known ICI targets, including CTLA-4, PD-1, PD-L1, IDO1, TIM-3, and PD-L2. We found that the risk Score was positively correlated with the expression of CTLA-4 and TIM-3 (Fig. 7D). Moreover, we analyzed the relationships between the risk signature and mutations. A higher number of mutations was observed in the high-risk group (Fig. 7E), and patients with HCC with *TP53* mutations showed higher risk scores than those without *TP53* mutations (Fig. 7F).

Furthermore, we analyzed the expression and role of the two risk genes in HCC. As shown in Figure S10A and S10B, the two risk genes were associated with a poor prognosis in TCGA and ICGC datasets. Single-cell sequencing analysis using the tSNE cluster web tool (mentioned previously in the Material and Methods section) also revealed *ATIC* and *OLA1* expression in immune cells. Figure S10C and D show that the two genes were expressed more abundantly in the C8_CD4-CTLA4, C4_CD8-LAYN, C5_CD8-GZMK, and C10_CD4-CXCL13 bundles of HCC tissues than in normal liver tissues. Immunohistochemistry analysis using the HPA database (https://www.proteinatlas.org/) further showed that OLA1 protein level was increased in HCC tissues (Figure S10E). No data for ATIC expression were available in the HPA database.

### Correlation between the risk model and drug sensitivity of HCC

**Fig. 8:**
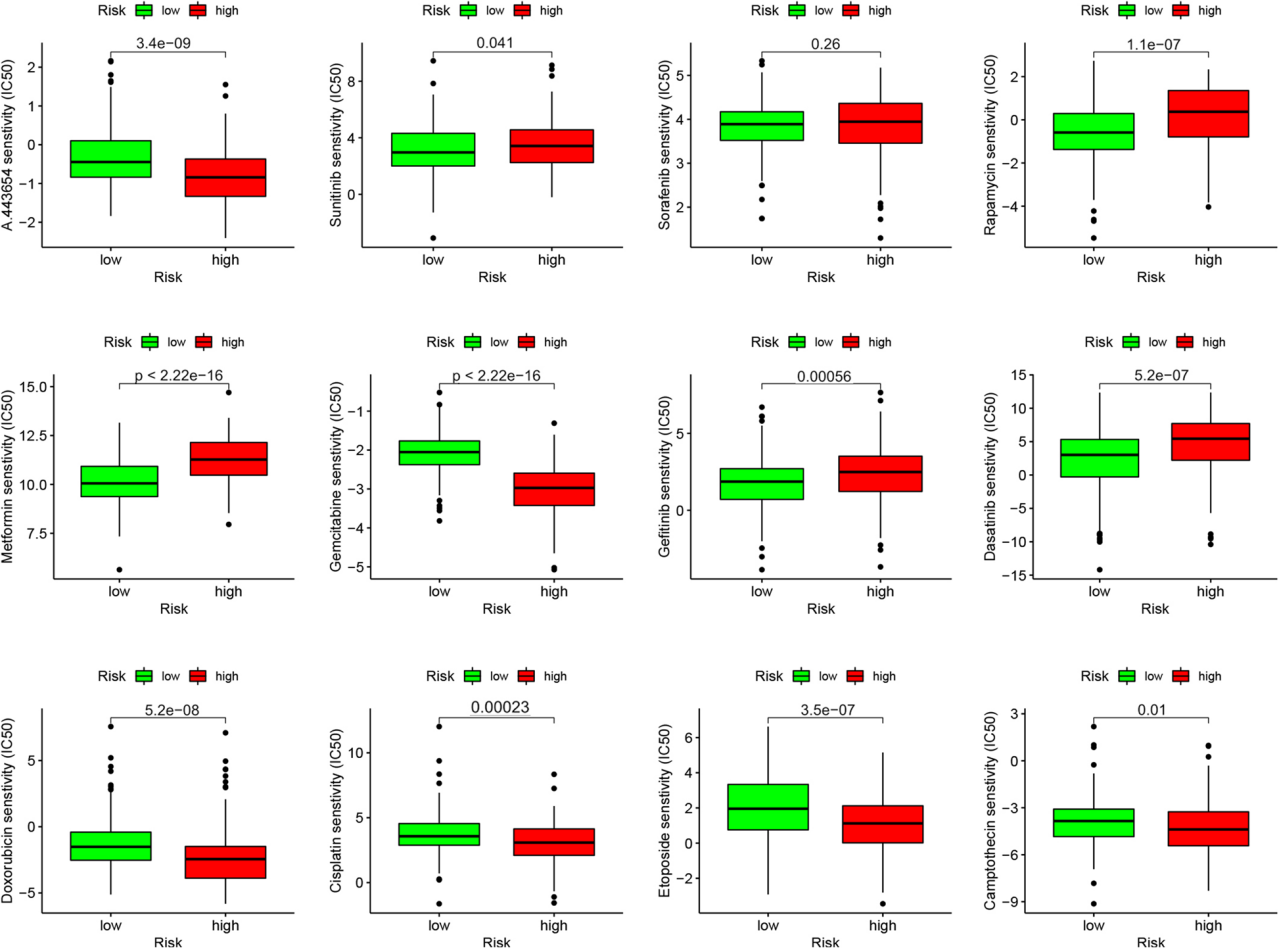
Drug sensitivity of HCC patients with high/low risk

In addition to ICI therapy, we identified the associations between the risk score and efficacy of common drugs that were used against HCC in TCGA-LIHC project dataset. The data showed that a high-risk score was associated with lower IC_50_ values for drugs such as etoposide (*P* < 0.001), A.443654 (*P* < 0.001), doxorubicin (*P* = 0.026), gemcitabine (*P* < 0.001), and cisplatin (*P* < 0.001) and with higher IC_50_ values for dasatinib (*P* < 0.001), gefitinib (*P* < 0.001), metformin (*P* < 0.001), and rapamycin (*P* < 0.001). These findings indicated that the model could be a predictor for drug sensitivity of HCC (Fig. 8).

## Discussion

Our comprehensive integrated analysis of M0RGs in HCC enhances the understanding of the molecular events relevant to HCC progression and treatment. The bioinformatics tools used in the current study have facilitated efficient prediction of the composition and changes in the TIME of HCC. The robust statistical power provided by relatively large sample sizes in TCGA and ICGC databases enabled the identification and validation of an M0RG prognostic signature. This is the first M0 macrophage-related risk score model for HCC; the model exhibited a good potential for the evaluation of HCC prognosis and the selection of a therapeutic strategy for HCC. Systematic analysis revealed that high risk scores were associated with a poor prognosis, immune infiltration, and gene mutations, and multivariate analysis confirmed that the risk model was an independent prognostic factor for patients with HCC.

Our results showed that the risk model was positively correlated with CTLA4, PD-L1, and TIM-3 expression, suggesting a potential role of the risk model in evaluating the efficacy of ICI therapy. The liver is the largest immune organ in the human body. Carcinogenic factors, such as persistent hepatitis B and C viral infections [17, 18], can compromise the immune defense or balance, rendering the immune cells unable to remove carcinogens [19, 20]. In early stages of tumor initiation, immune suppression decreases immune surveillance [21]. Thus, ICIs, such as PD-1/PD-L1 inhibitors, have become a promising treatment for HCC as they activate and restore immune functions for the optimal ablation of tumor cells [22–24]. Identifying a predictive model is of great importance for improving HCC immune therapy. We identified the associations between the risk model and drug sensitivity in HCC. A high-risk score was associated with lower IC_50_ values of several drugs, which indicated that this model could be a predictor for drug sensitivity in HCC as a clinical reference. For example, the potential antitumor effects of metformin can be further investigated using the “new uses of old drugs” strategy for drug repositioning.

The significance of our study lies not only in the revelation of the composition of infiltrating immune cells in HCC but also in the demonstration of a systematic association of the M0 phenotype and gene clusters with genomic characteristics and clinical features. To this end, we identified biomarkers for potential clinical application. These biomarkers were further used to construct a risk model to predict the prognosis of patients with HCC. Analysis of TCGA datasets revealed that M0 macrophages and relevant genes were unfavorable factors that correlated with clinical features and prognosis of HCC. These results contrasted, to some extent, with previous findings, which suggested that the differentiation of polarized M1 or M2 macrophages was associated with functional properties of tumors [25, 26]. The canonical M1 versus M2 dichotomy has been challenged by recent evidence supporting abundant differentiation of nonpolarized M0 macrophages, rather than that of M1 or M2 macrophages, in tumors [11, 12]. M0 macrophages are defined as undifferentiated macrophages with the potential to polarize into specific macrophage subtypes. Different subtypes of liver macrophages, especially Kupffer cells and TAMs, exhibit diverse ontogeny, differentiation, and function [27, 28]. TAMs have been significantly implicated in HCC initiation, progression, immune evasion, invasion, angiogenesis, and metastasis, as well as in response to therapy [29]. Liver macrophages exhibit highly variable phenotypes that are modulated by signals derived from the liver microenvironment. M1 and M2 macrophages coexist in the tumor microenvironment of various cancers, which may be because of a continuous, rather than isolated, process of M0 macrophage polarization into M1 and M2 macrophages [30]. Based on our findings, it is hypothesized that the infiltration and differentiation of TAMs in the liver are possibly stimulated in response to carcinogenic factors, thus promoting chronic inflammation, suppressing immunity, and leading to HCC progression.

Single-cell analysis of infiltrating immune cells allows in-depth understanding of the landscape of these cells in the highly complicated tumor microenvironment. Recently, single-cell transcriptome technology has been applied to cancerous and immune cells from patients with HCC, resulting in the identification of 11 T cell subsets based on their molecular and functional properties, which delineate their developmental trajectory [31]. In the present study, we analyzed the expression of M0RGs using single-cell sequencing data, and the results revealed that two M0RGs, *ATIC* and *OLA1*, were expressed more abundantly in specific subgroups of T cells with signature markers, such as the CD4-CTLA4, CD8-LAYN, CD8-GZMK, and CD4-CXCL13 bundles of HCC tissues. Based on these results, we can propose two scientific hypotheses. First, these specific subgroups might be activated in the HCC microenvironment. The status of T cell infiltration and their characteristics are usually associated with different prognostic outcomes [32]. Several studies have also revealed the association of LAYN, CTLA4, and GZMK expression with tumor-infiltrating exhausted CD8^+^ T cells and a poor prognosis [31]. Therefore, inhibiting these specific cells might be another strategy for cancer immunotherapy. Second, there may be a crosstalk between macrophages and T cells in the HCC microenvironment, which plays a role in influencing HCC progression and therapeutic efficacy. The polarization and function of HCC-associated macrophages are possibly regulated via these specific subgroups of T cells, which still requires further elucidation.

To our knowledge, this is the first study to construct a risk model based on M0RGs in HCC; the model showed that a low-risk score reflected a good prognosis, whereas a high-risk score indicated a poor prognosis, suggesting that the risk model is a robust prognostic biomarker. Further analysis revealed that cancer- and immune-related signaling pathways were enriched in the high-risk group. B memory cells, Tregs, and M0 macrophages were positively associated with the risk score. These results are in agreement with the prevailing knowledge that pathological division of cells is the basis of tumorigenesis and that immune tolerance can facilitate tumor development [30, 33]. Additionally, we observed that our risk model was associated with known somatic mutations in *TP53*. These alterations in a somatic gene may inactivate tumor suppressor genes and cause mutations in protooncogenes, resulting in tumorigenesis [34]. Therefore, our study contributes to the identification of immunotherapeutic targets to inhibit the pathways involved in tumorigenesis.

The major limitation of our study is the lack of biological validation of immune cell infiltration in vitro and in vivo in HCC tissues because of a delayed arrival of antibodies due to the coronavirus disease pandemic. Unlike other studies on immune infiltration in HCC [35], our analysis mainly focused on M0 macrophages using a large number of HCC samples from public databases. In addition to the function of immune cells in the TIME, we comprehensively mapped the landscape of interactions involving M0 macrophage-associated immune cells, genes, and clinicopathological features. Moreover, we confirmed that the prognostic value of the risk model was superior to that of other clinical signatures. This precise and simple model of two M0RGs will contribute to evaluating the prognosis of and treatment efficacy in HCC. We also revealed promising immune-based candidate biomarkers for the diagnosis, prognosis, and therapy of HCC.

## Conclusions

In conclusion, our comprehensive integrated analysis of M0RGs in HCC enhances the understanding of molecular events relevant to HCC progression and treatment. A risk model was constructed based on M0RGs and validated in clinical cohorts, which exhibited robust prognostic value for patients with HCC. We also revealed promising candidate immune-based biomarkers for diagnosis, prognosis, and therapy in HCC.

## Supplementary Information

**Additional file 1: TableS1.** Macrophage_M0 related genes using Pearson’sanalysis. **Table S2.** M0RGs associatedwith the prognosis of patients with HCC in TCGA datasets using univariate Coxanalysis. **Table S3.** M0RGs associatedwith the prognosis of patients with HCC in ICGC datasets using univariate Coxanalysis. **Figure S1.** M0 macrophages inHCC. **Figure S2.** The CNV and mutationstatus of 35 M0RGs. A, The CNV status of 35 M0RGs. B, The mutation status of 35M0RGs. **Figure S3.** The best cutoff value to distinguish the high-and low-risk groups. **Figure S4.** Prognostic model of the test (GSE14520) cohort. Riskscore of the high and low groups. Heatmap of the expression of 2 M0RGs.Survival analysis of the high and low groups. The AUC of the ROC. M0RGs: M0macrophages-related genes; AUC: Area under curve; ROC: Receiver operatingcharacteristic curve. **Figure S5.** Thesurvival analysis of OLA1 were analyzed using the X-Tile software. **Figure S6.** The survival analysis ofATIC were analyzed using the X-Tile software. **Figure S7.**C-index for discrimination. **Figure S8.** The HCC patients with highrisk also had poor prognosis of OS with different clinical characters. **Figure S9.** Construction of the nomogram in the ICGC dataset. Thenomogram to predict the 3-year survival risk of HCC patients. **Figure S10.** The expression andprognosis of M0RGs in HCC. A, The survival analysis of HCC with high/low ATICand OLA1 in TCGA. B, The survival analysis of HCC with high/low ATIC and OLA1in ICGC. C, ATIC and D, OLA1 expression in immune cells using tSNE cluster webtool. E, The protein levels of OLA1 in HCC using HPA dataset. **Figure S11.** Schematic depictingthe construction of an M0 macrophage-related prognostic model forhepatocellular carcinoma.

## Data Availability

All data generated or analyzed during this study are included in the published article.

## Abbreviations

ATIC: 5-Aminoimidazole-4-carboxamide ribonucleotide formyl transferase/inosine monophosphate cyclohydrolase
AUC: Area under the curve
CNV: Copy number variation
DFI: Disease-free interval
DSS: Disease-associated survival
GSEA: Gene set enrichment analysis
HCC: Hepatocellular carcinoma
IC_50_: Half-maximal inhibitory concentration
ICGC: International Cancer Genome Consortium
ICI: Immune checkpoint inhibitor
LIHC: Liver hepatocellular carcinoma
M0RG: M0 macrophage-related gene
OLA1: Obg-like ATPase 1
OS: Overall survival
PFI: Progression-free interval
ROC: Receiver operating characteristic
TAM: Tumor-associated macrophage
TCGA: The Cancer Genome Atlas
TIME: Tumor immune microenvironment
Treg: Regulatory T cell
