# Supplementary material for: An M0 macrophage-related prognostic model for hepatocellular carcinoma
Source: BMC Cancer. 2022 Jul 19;22:791. doi: 10.1186/s12885-022-09872-y (PMC9294844; doi:10.1186/s12885-022-09872-y)
Supplement: Supplementary file 1 — Additional file 1: TableS1. Macrophage_M0 related genes using Pearson’sanalysis. Table S2. M0RGs associatedwith the prognosis of patients with HCC in TCGA datasets using univariate Coxanalysis. Table S3. M0RGs associatedwith the prognosis of patients with HCC in ICGC datasets using univariate Coxanalysis. Figure S1. M0 macrophages inHCC. Figure S2. The CNV and mutationstatus of 35 M0RGs. A, The CNV status of 35 M0RGs. B, The mutation status of 35M0RGs. Figure S3. The best cutoff value to distinguish the high-and low-risk groups. Figure S4. Prognostic model of the test (GSE14520) cohort. Riskscore of the high and low groups. Heatmap of the expression of 2 M0RGs.Survival analysis of the high and low groups. The AUC of the ROC. M0RGs: M0macrophages-related genes; AUC: Area under curve; ROC: Receiver operatingcharacteristic curve. Figure S5. Thesurvival analysis of OLA1 were analyzed using the X-Tile software. Figure S6. The survival analysis ofATIC were analyzed using the X-Tile software. Figure S7.C-index for discrimination. Figure S8. The HCC patients with highrisk also had poor prognosis of OS with different clinical characters. Figure S9. Construction of the nomogram in the ICGC dataset. Thenomogram to predict the 3-year survival risk of HCC patients. Figure S10. The expression andprognosis of M0RGs in HCC. A, The survival analysis of HCC with high/low ATICand OLA1 in TCGA. B, The survival analysis of HCC with high/low ATIC and OLA1in ICGC. C, ATIC and D, OLA1 expression in immune cells using tSNE cluster webtool. E, The protein levels of OLA1 in HCC using HPA dataset. Figure S11. Schematic depictingthe construction of an M0 macrophage-related prognostic model forhepatocellular carcinoma. [file 12885_2022_9872_MOESM1_ESM.doc]

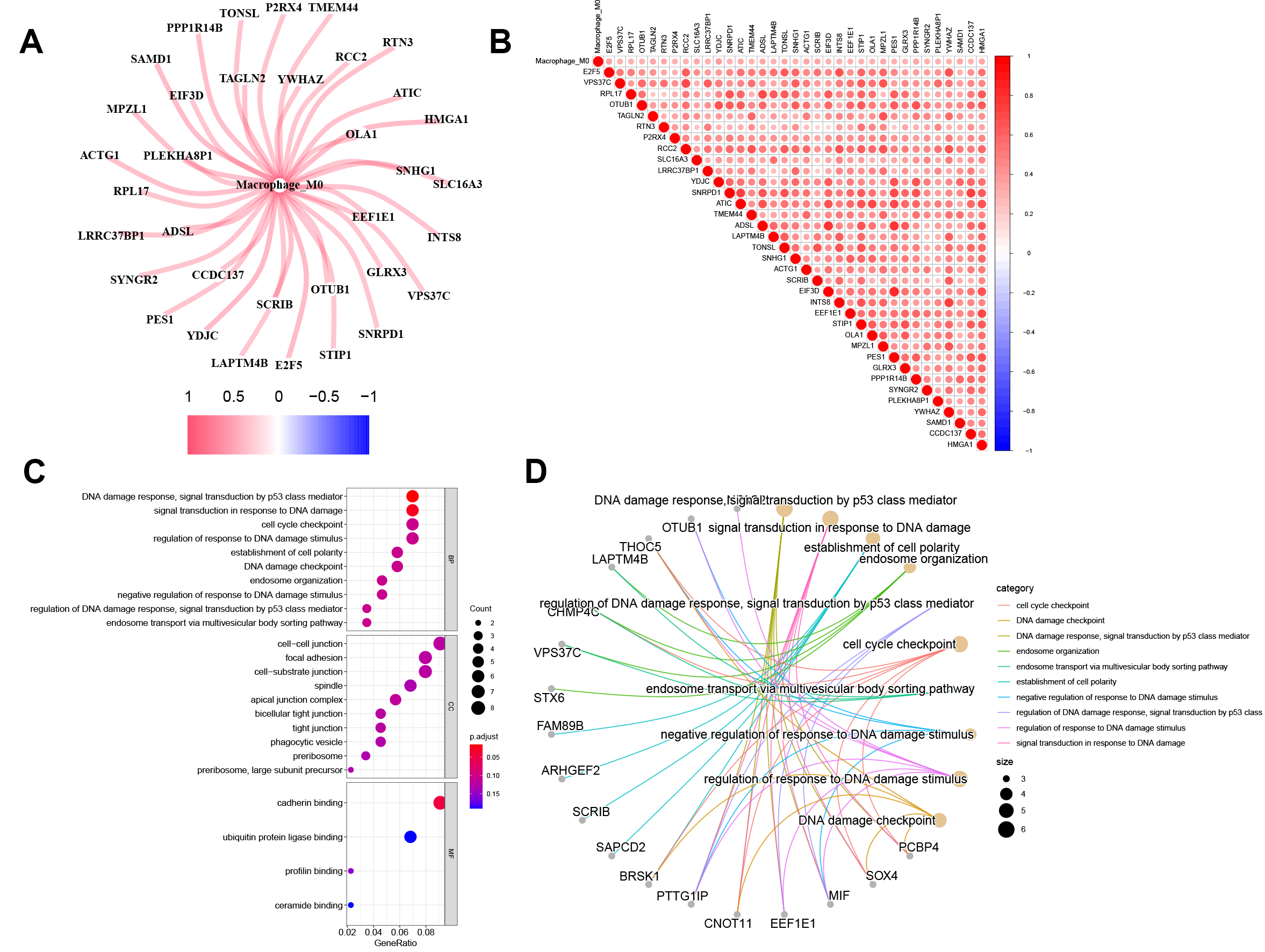


Figure 1. M0 macrophages related genes in HCC. A and B, A correlation network involving the 35 prognosis-related M0RGs and M0 macrophages in the TCGA cohort. C and D, GO analyzed of the 35 M0RGs.

M0RGs: M0 macrophages-related genes; TCGA: The Cancer Genome Atlas; Go: Gene Ontology.


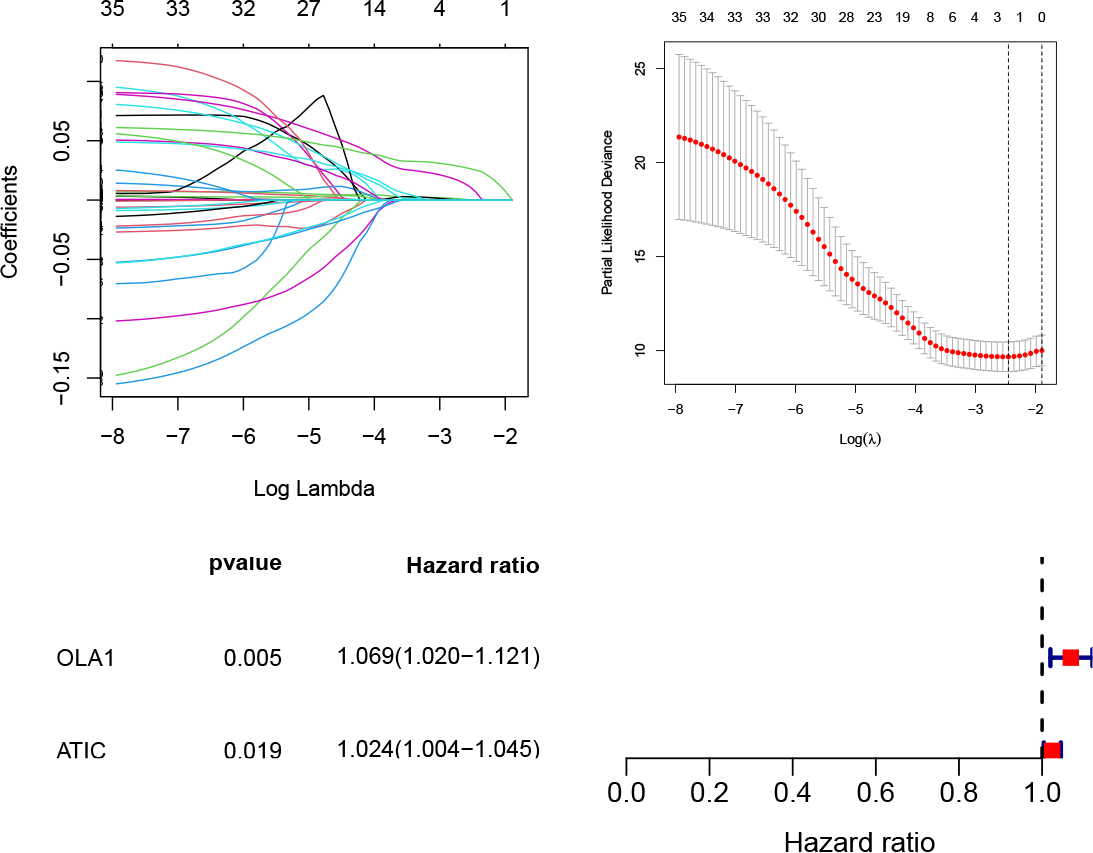


Figure 2. The M0RGs prognostic signature. A, Cross-validation for tuning parameter (lambda, screening in the LASSO regression model. B, LASSO coefﬁcient proﬁles of 35 prognostic M0RGs. C, Forest plot of the seven DNA replication-related genes.

M0RGs: M0 macrophages-related genes


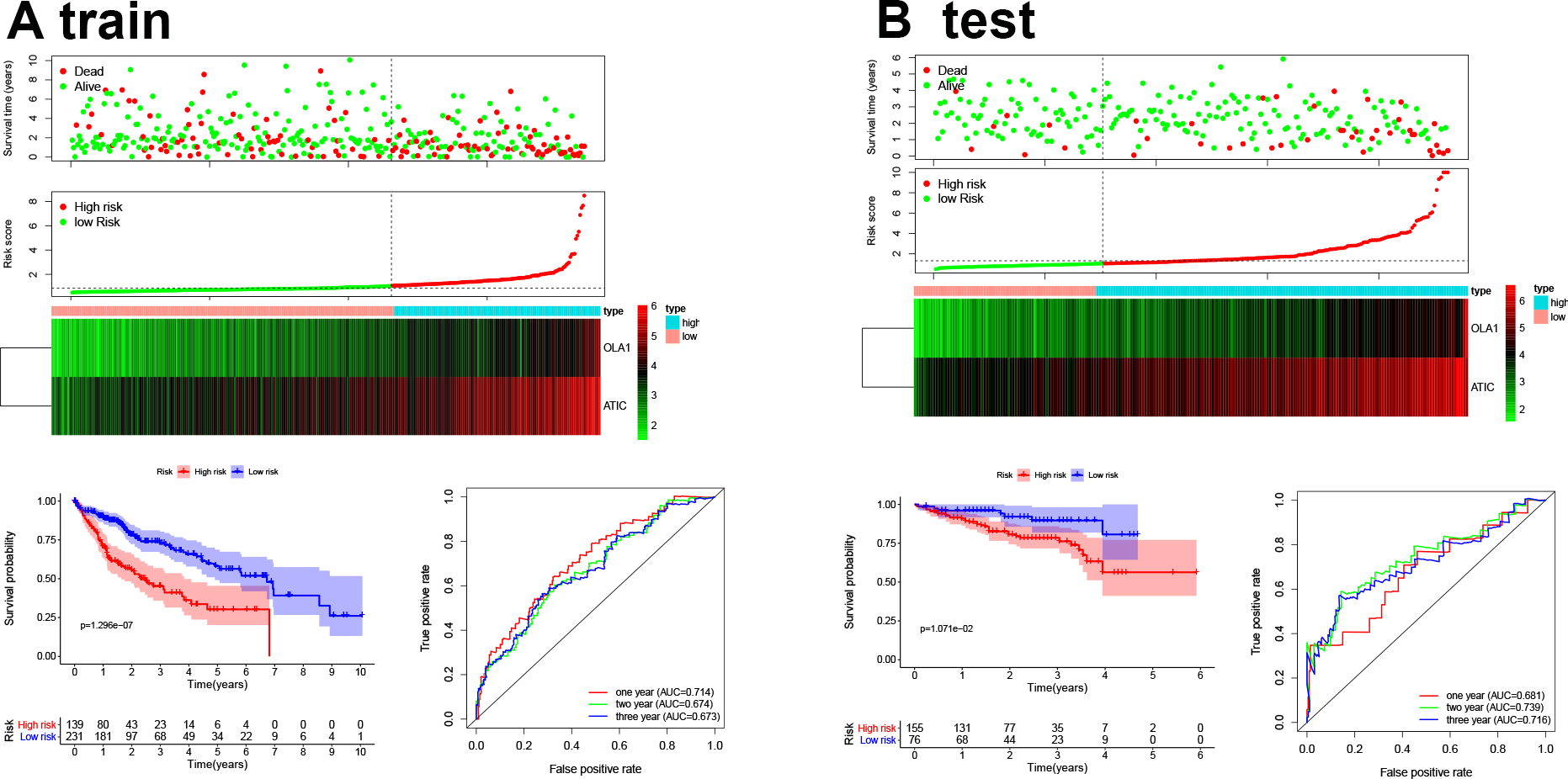


Figure 3. Prognostic model of the train (TCGA) cohort and test (ICGC) cohort. (A) Train set (B) Test set. Risk score of the high and low groups. Heatmap of the expression of 2 M0RGs. Survival analysis of the high and low groups. The AUC of the ROC.

TCGA: The Cancer Genome Atlas; ICGC: International Cancer Genome Consortium; M0RGs: M0 macrophages-related genes; AUC: Area under curve; ROC: Receiver operating characteristic curve


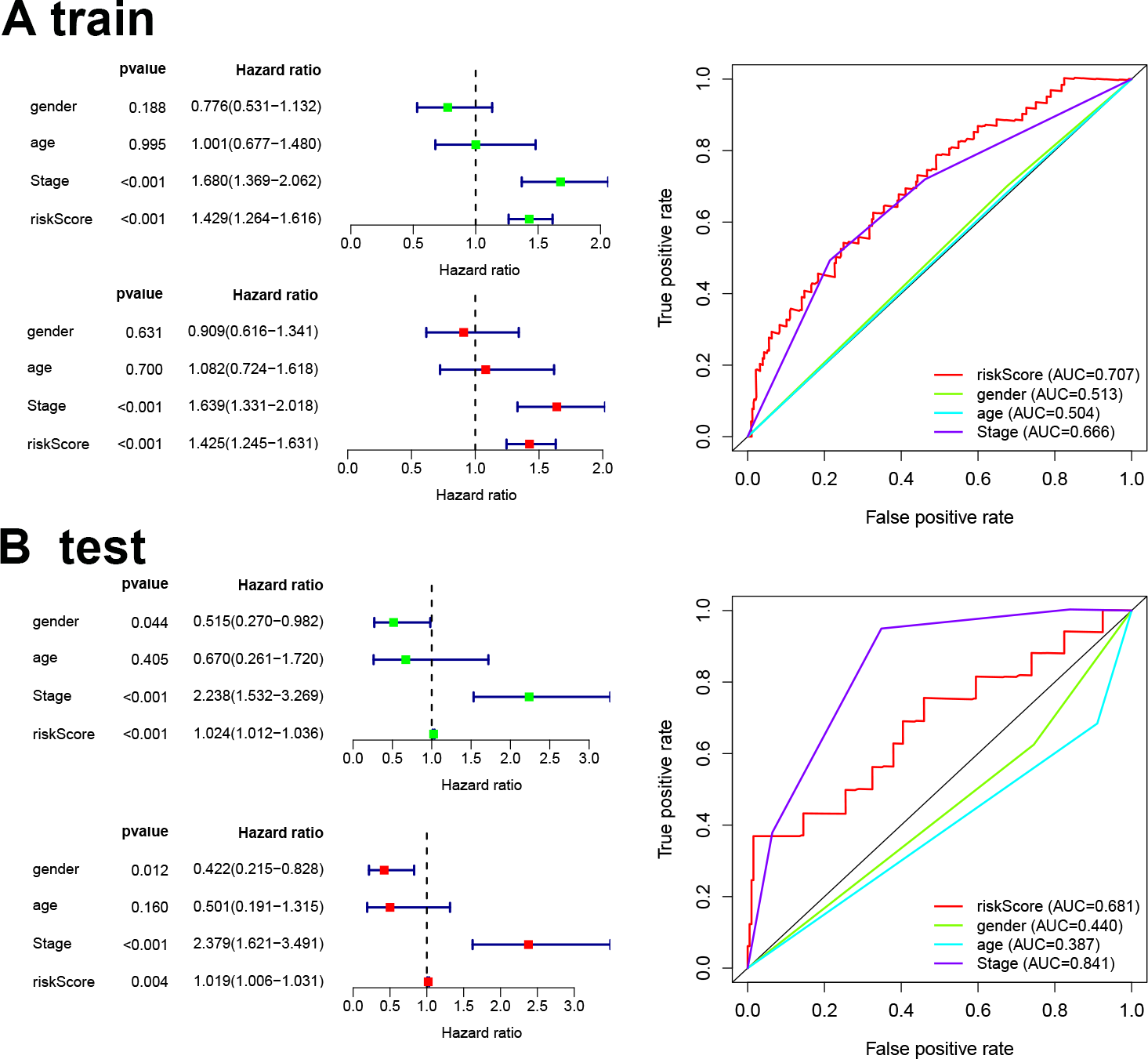


Figure 4. Association between the clinicopathological characteristics and prognostic risk score. A, Univariate and multivariate Cox regression analyses and ROC value in training group. B, Univariate and multivariate Cox regression analyses and ROC value in testing group.

ROC: Receiver operating characteristic curve


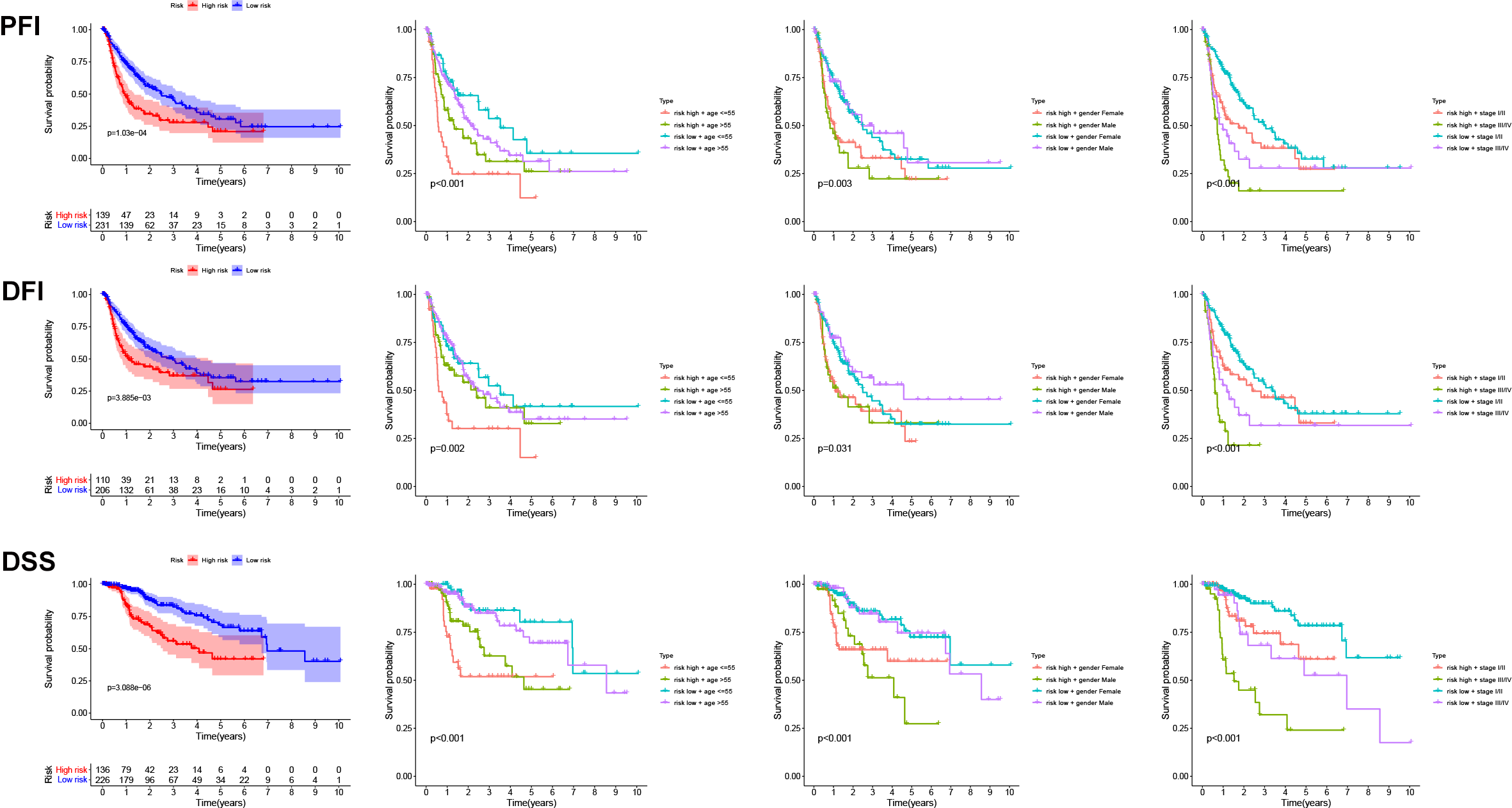


Figure 5. The prognosis of HCC patients with high/low risk score.

HCC: Hepatocellular carcinoma


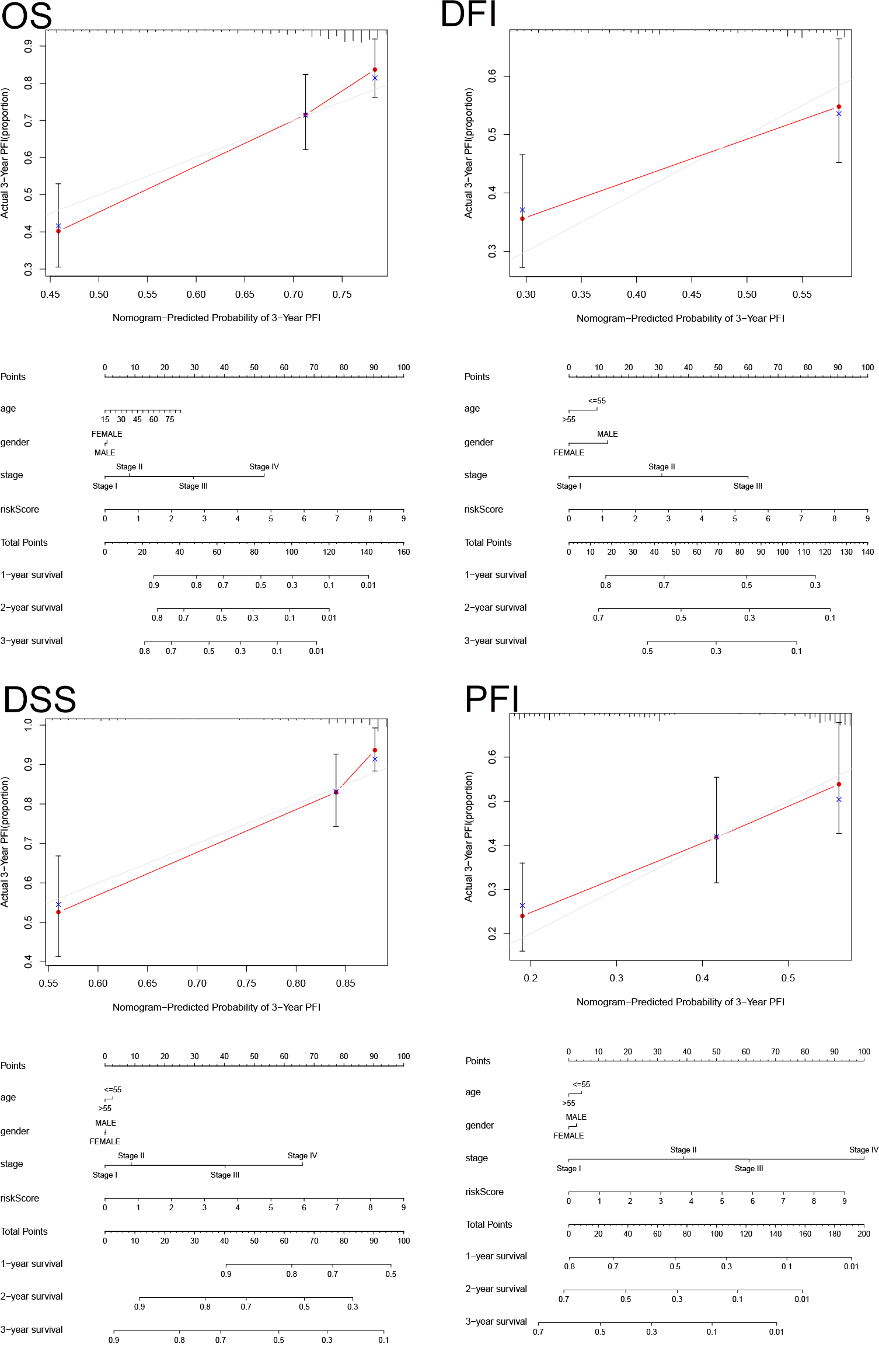


Figure 6. Construction of the nomogram in the TCGA dataset. The nomogram to predict the 1-, 2- and 3-year survival risk of HCC patients. The calibration curve of the 3-year survival.


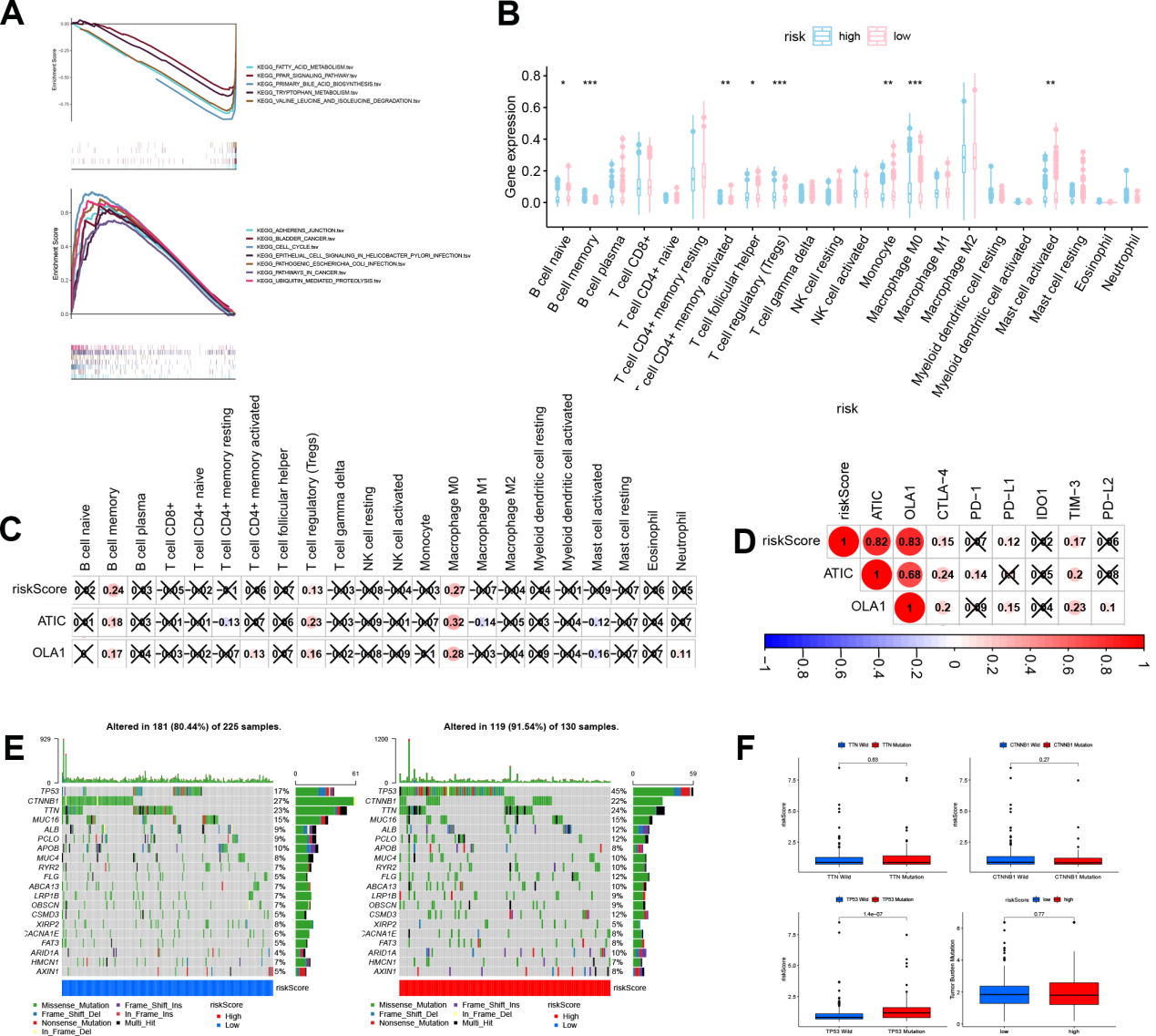


Figure 7. The Relationship between M0RGs and Immune infiltration, mutation state. A, GSEA analysis. B, immune cell in high/low groups. C, The relationship between immune cell and risk score. D, The relationship between risk score and ICB. E, The mutation in high/low risk group. F, The relationship between risk score and TP53, TTN, CTNNB1 mutation, TMB.


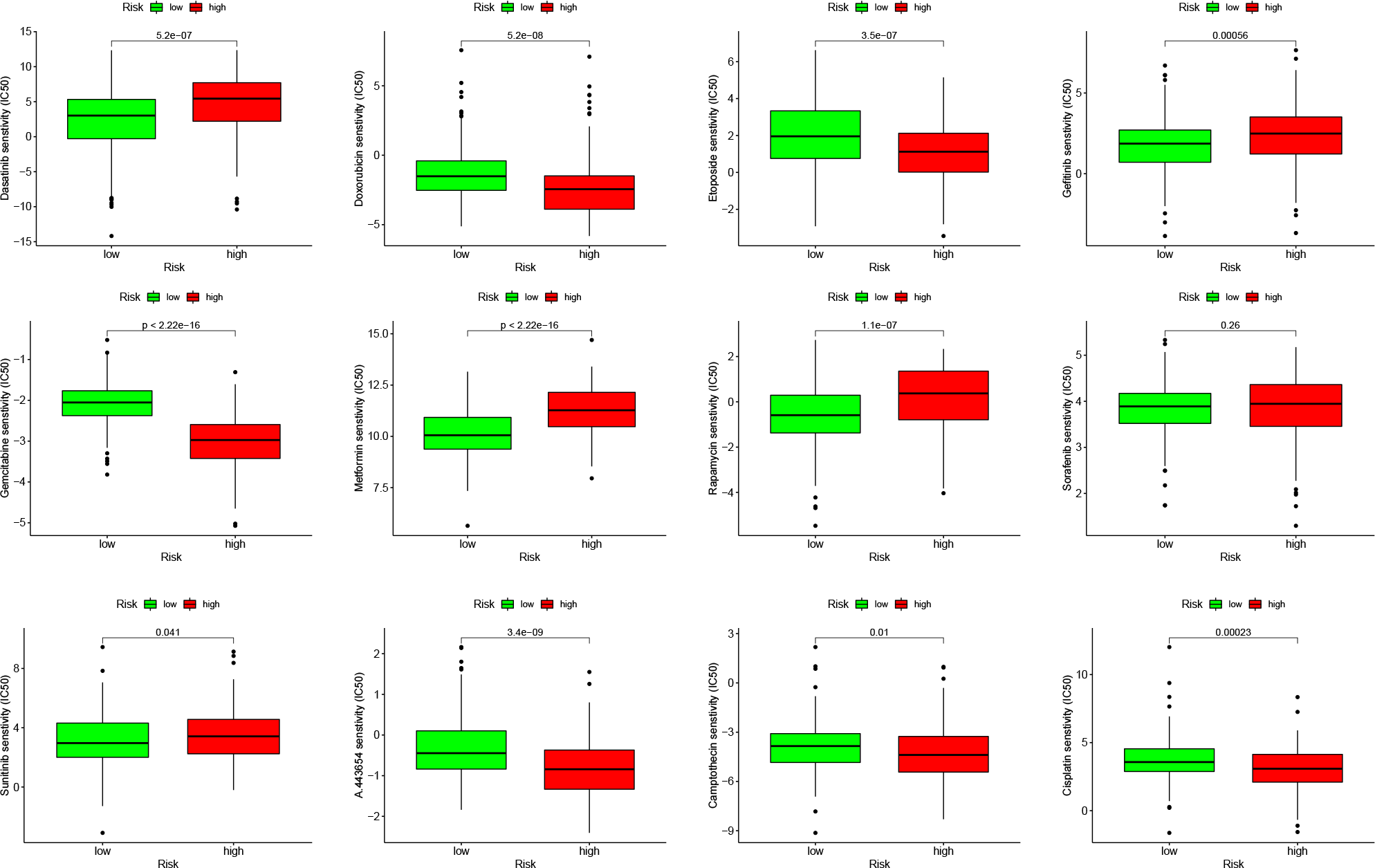


Figure 8 Drug sensitivity of HCC patients with high/low risk.

Table S1: Macrophage_M0 related genes using Pearson’s analysis.

| gene1 | gene2 | cor | pvalue |
| --- | --- | --- | --- |
| Macrophage_M0 | Macrophage_M0 | 1 | 0 |
| Macrophage_M0 | MMP9 | 0.463 | 5.20E-21 |
| Macrophage_M0 | MEX3A | 0.421 | 2.68E-17 |
| Macrophage_M0 | FAM241B | 0.396 | 2.33E-15 |
| Macrophage_M0 | ARHGEF2 | 0.374 | 9.74E-14 |
| Macrophage_M0 | YDJC | 0.364 | 5.02E-13 |
| Macrophage_M0 | E2F5 | 0.356 | 1.80E-12 |
| Macrophage_M0 | ENAH | 0.35 | 4.42E-12 |
| Macrophage_M0 | SAMD1 | 0.35 | 3.99E-12 |
| Macrophage_M0 | HMGA1 | 0.35 | 4.33E-12 |
| Macrophage_M0 | ZNF239 | 0.349 | 5.25E-12 |
| Macrophage_M0 | CSTB | 0.346 | 7.82E-12 |
| Macrophage_M0 | RANGAP1 | 0.344 | 1.06E-11 |
| Macrophage_M0 | FHL3 | 0.343 | 1.25E-11 |
| Macrophage_M0 | PPP1R14B | 0.342 | 1.32E-11 |
| Macrophage_M0 | ACTG1 | 0.336 | 3.06E-11 |
| Macrophage_M0 | ZNF485 | 0.335 | 3.75E-11 |
| Macrophage_M0 | TAGLN2 | 0.331 | 6.75E-11 |
| Macrophage_M0 | MIF | 0.331 | 6.75E-11 |
| Macrophage_M0 | CNOT11 | 0.331 | 6.99E-11 |
| Macrophage_M0 | ADSL | 0.328 | 9.49E-11 |
| Macrophage_M0 | MPZL1 | 0.328 | 9.82E-11 |
| Macrophage_M0 | SLC16A3 | 0.327 | 1.07E-10 |
| Macrophage_M0 | ATIC | 0.327 | 1.16E-10 |
| Macrophage_M0 | CAPN10 | 0.326 | 1.25E-10 |
| Macrophage_M0 | SOX4 | 0.326 | 1.32E-10 |
| Macrophage_M0 | SCRIB | 0.326 | 1.27E-10 |
| Macrophage_M0 | RTN3 | 0.324 | 1.66E-10 |
| Macrophage_M0 | AC005332.5 | 0.324 | 1.83E-10 |
| Macrophage_M0 | PLPP2 | 0.323 | 2.01E-10 |
| Macrophage_M0 | CAPG | 0.323 | 1.95E-10 |
| Macrophage_M0 | TONSL | 0.322 | 2.16E-10 |
| Macrophage_M0 | ANXA11 | 0.322 | 2.32E-10 |
| Macrophage_M0 | AKAP8L | 0.322 | 2.11E-10 |
| Macrophage_M0 | TRAF4 | 0.318 | 4.06E-10 |
| Macrophage_M0 | PPP1R14BP3 | 0.318 | 4.11E-10 |
| Macrophage_M0 | AC068473.5 | 0.316 | 4.90E-10 |
| Macrophage_M0 | SNU13 | 0.316 | 5.26E-10 |
| Macrophage_M0 | UNC119 | 0.315 | 5.49E-10 |
| Macrophage_M0 | INTS8 | 0.315 | 5.94E-10 |
| Macrophage_M0 | HMGXB3 | 0.315 | 5.45E-10 |
| Macrophage_M0 | ZNF623 | 0.315 | 6.01E-10 |
| Macrophage_M0 | TRMU | 0.314 | 6.19E-10 |
| Macrophage_M0 | LRRC37BP1 | 0.314 | 6.31E-10 |
| Macrophage_M0 | SLC52A2 | 0.314 | 6.96E-10 |
| Macrophage_M0 | BRSK1 | 0.313 | 7.79E-10 |
| Macrophage_M0 | CYTH2 | 0.313 | 7.09E-10 |
| Macrophage_M0 | AP001453.3 | 0.312 | 8.86E-10 |
| Macrophage_M0 | PES1 | 0.312 | 8.77E-10 |
| Macrophage_M0 | PLEKHA8P1 | 0.311 | 9.97E-10 |
| Macrophage_M0 | PTTG1IP | 0.311 | 1.02E-09 |
| Macrophage_M0 | CFAP298 | 0.31 | 1.18E-09 |
| Macrophage_M0 | P2RX4 | 0.31 | 1.15E-09 |
| Macrophage_M0 | TMEM68 | 0.309 | 1.25E-09 |
| Macrophage_M0 | RPL17 | 0.309 | 1.20E-09 |
| Macrophage_M0 | SYNGR2 | 0.309 | 1.22E-09 |
| Macrophage_M0 | SAPCD2 | 0.308 | 1.48E-09 |
| Macrophage_M0 | STIP1 | 0.308 | 1.47E-09 |
| Macrophage_M0 | YWHAZ | 0.308 | 1.42E-09 |
| Macrophage_M0 | CCDC137 | 0.308 | 1.38E-09 |
| Macrophage_M0 | CEP164 | 0.308 | 1.34E-09 |
| Macrophage_M0 | POP1 | 0.308 | 1.45E-09 |
| Macrophage_M0 | VPS37C | 0.307 | 1.61E-09 |
| Macrophage_M0 | OTUB1 | 0.307 | 1.64E-09 |
| Macrophage_M0 | SNRPD1 | 0.307 | 1.70E-09 |
| Macrophage_M0 | EEF1E1 | 0.307 | 1.57E-09 |
| Macrophage_M0 | THOC5 | 0.307 | 1.66E-09 |
| Macrophage_M0 | FAM89B | 0.307 | 1.61E-09 |
| Macrophage_M0 | NPAS2 | 0.306 | 1.80E-09 |
| Macrophage_M0 | TRMT2A | 0.306 | 1.80E-09 |
| Macrophage_M0 | CHMP4C | 0.306 | 1.83E-09 |
| Macrophage_M0 | SPHK1 | 0.306 | 1.80E-09 |
| Macrophage_M0 | STX6 | 0.305 | 2.16E-09 |
| Macrophage_M0 | TTLL1 | 0.305 | 2.14E-09 |
| Macrophage_M0 | NPM3 | 0.305 | 2.02E-09 |
| Macrophage_M0 | DPAGT1 | 0.304 | 2.43E-09 |
| Macrophage_M0 | RRP1 | 0.304 | 2.40E-09 |
| Macrophage_M0 | PCBP4 | 0.304 | 2.44E-09 |
| Macrophage_M0 | OLA1 | 0.304 | 2.51E-09 |
| Macrophage_M0 | SGSM3 | 0.304 | 2.29E-09 |
| Macrophage_M0 | LAPTM4B | 0.303 | 2.55E-09 |
| Macrophage_M0 | SNHG1 | 0.303 | 2.56E-09 |
| Macrophage_M0 | BACE1-AS | 0.303 | 2.87E-09 |
| Macrophage_M0 | GLRX3 | 0.303 | 2.64E-09 |
| Macrophage_M0 | TMEM65 | 0.303 | 2.56E-09 |
| Macrophage_M0 | HOMER3 | 0.302 | 3.22E-09 |
| Macrophage_M0 | NEURL3 | 0.302 | 3.21E-09 |
| Macrophage_M0 | EIF3D | 0.302 | 2.91E-09 |
| Macrophage_M0 | AC003072.1 | 0.302 | 2.89E-09 |
| Macrophage_M0 | ZNF74 | 0.301 | 3.65E-09 |
| Macrophage_M0 | RCC2 | 0.301 | 3.45E-09 |
| Macrophage_M0 | ZNF250 | 0.301 | 3.32E-09 |
| Macrophage_M0 | ANKRD13D | 0.301 | 3.47E-09 |
| Macrophage_M0 | TMEM44 | 0.301 | 3.42E-09 |
| Macrophage_M0 | ARHGAP39 | 0.301 | 3.43E-09 |
| Macrophage_M0 | NCF2 | 0.301 | 3.48E-09 |
| Macrophage_M0 | C11orf80 | 0.301 | 3.54E-09 |
| Macrophage_M0 | ITIH1 | -0.315 | 5.39E-10 |
| Macrophage_M0 | IVD | -0.317 | 4.69E-10 |
| Macrophage_M0 | TMEM220-AS1 | -0.321 | 2.71E-10 |

Table S2: M0RGs associated with the prognosis of patients with HCC in TCGA datasets using univariate Cox analysis.

| id | HR | HR.95L | HR.95H | pvalue |
| --- | --- | --- | --- | --- |
| STX6 | 1.189501709 | 1.10243539 | 1.283444208 | 7.66E-06 |
| HOMER3 | 1.026225965 | 1.000479354 | 1.052635146 | 0.045832647 |
| E2F5 | 1.39320644 | 1.210827621 | 1.603055753 | 3.61E-06 |
| AC068473.5 | 1.407409573 | 1.179751662 | 1.678998869 | 0.000146959 |
| DPAGT1 | 1.072988582 | 1.026149252 | 1.121965926 | 0.001978403 |
| BRSK1 | 1.593766806 | 1.249385838 | 2.033073016 | 0.000175078 |
| CFAP298 | 1.095972428 | 1.000612885 | 1.200419844 | 0.048477787 |
| VPS37C | 1.172007834 | 1.083315333 | 1.267961711 | 7.71E-05 |
| NPAS2 | 1.189779583 | 1.062507533 | 1.332296867 | 0.002609534 |
| SNU13 | 1.016821512 | 1.00503708 | 1.028744121 | 0.005035686 |
| FAM241B | 1.058689449 | 1.027788061 | 1.090519916 | 0.000161004 |
| TTLL1 | 1.23818303 | 1.114088511 | 1.376100014 | 7.34E-05 |
| AP001453.3 | 1.60081813 | 1.135722172 | 2.256378142 | 0.007216626 |
| RPL17 | 1.024241407 | 1.004894617 | 1.043960673 | 0.013824022 |
| TMEM220-AS1 | 0.759681756 | 0.655367462 | 0.880599671 | 0.000265093 |
| OTUB1 | 1.048169782 | 1.014812493 | 1.082623538 | 0.004357604 |
| TRMT2A | 1.063661866 | 1.022570609 | 1.106404344 | 0.002138285 |
| TAGLN2 | 1.002442101 | 1.001302844 | 1.003582654 | 2.62E-05 |
| RTN3 | 1.035848698 | 1.022200974 | 1.049678636 | 1.94E-07 |
| P2RX4 | 1.092349754 | 1.024470548 | 1.164726491 | 0.006964274 |
| RANGAP1 | 1.010561462 | 1.002481911 | 1.018706131 | 0.010311441 |
| ZNF74 | 1.381931671 | 1.132787089 | 1.685872977 | 0.001426753 |
| RCC2 | 1.03396195 | 1.018455277 | 1.049704723 | 1.48E-05 |
| PLPP2 | 1.027164196 | 1.007844962 | 1.046853758 | 0.005664525 |
| ITIH1 | 0.998338113 | 0.997416163 | 0.999260914 | 0.000418042 |
| TRMU | 1.128194696 | 1.042706292 | 1.220692041 | 0.002698584 |
| ZNF250 | 1.494474376 | 1.048872112 | 2.129386066 | 0.026141583 |
| SLC16A3 | 1.048170957 | 1.023332999 | 1.073611773 | 0.000120553 |
| CAPN10 | 1.62423325 | 1.337380431 | 1.97261272 | 9.98E-07 |
| ANKRD13D | 1.116698413 | 1.021990519 | 1.22018289 | 0.014645698 |
| UNC119 | 1.066813407 | 1.008218142 | 1.128814092 | 0.024837176 |
| LRRC37BP1 | 1.455253213 | 1.156989157 | 1.830407747 | 0.00134564 |
| YDJC | 1.0454594 | 1.014600978 | 1.077256361 | 0.003635008 |
| NEURL3 | 1.011107363 | 1.002067232 | 1.020229049 | 0.015924639 |
| SNRPD1 | 1.04580353 | 1.016784141 | 1.075651143 | 0.001813076 |
| AC005332.5 | 1.175550069 | 1.058320562 | 1.305765015 | 0.002548695 |
| ZNF239 | 1.346103834 | 1.210416822 | 1.497001281 | 4.19E-08 |
| ATIC | 1.042070578 | 1.026941832 | 1.057422199 | 3.33E-08 |
| SAPCD2 | 1.217176469 | 1.13298579 | 1.307623245 | 7.70E-08 |
| PCBP4 | 1.04103139 | 1.004700979 | 1.078675523 | 0.026504564 |
| TMEM44 | 1.173220429 | 1.056138475 | 1.303281915 | 0.002899332 |
| ENAH | 1.047596405 | 1.020419113 | 1.075497523 | 0.000525913 |
| ADSL | 1.071464328 | 1.041673851 | 1.102106774 | 1.60E-06 |
| LAPTM4B | 1.004234764 | 1.001736154 | 1.006739606 | 0.000885067 |
| ARHGAP39 | 1.186259097 | 1.085067987 | 1.296887073 | 0.000173594 |
| TONSL | 1.164788673 | 1.092159217 | 1.242248045 | 3.42E-06 |
| SNHG1 | 1.041783556 | 1.014642157 | 1.069650979 | 0.002372033 |
| SOX4 | 1.025735854 | 1.011285806 | 1.040392376 | 0.000447573 |
| ACTG1 | 1.001040691 | 1.000479251 | 1.001602445 | 0.00027919 |
| SCRIB | 1.019054451 | 1.001787044 | 1.036619488 | 0.030408282 |
| ARHGEF2 | 1.084444833 | 1.038603329 | 1.132309673 | 0.000234366 |
| EIF3D | 1.013143781 | 1.005124522 | 1.021227021 | 0.001279048 |
| INTS8 | 1.227223581 | 1.126145354 | 1.337374178 | 3.03E-06 |
| HMGXB3 | 1.176572239 | 1.095400303 | 1.263759221 | 8.26E-06 |
| EEF1E1 | 1.085628259 | 1.031347893 | 1.142765428 | 0.001692781 |
| STIP1 | 1.030920216 | 1.020661658 | 1.041281882 | 2.40E-09 |
| OLA1 | 1.108721468 | 1.071164655 | 1.147595084 | 4.36E-09 |
| CYTH2 | 1.084098154 | 1.020493344 | 1.151667294 | 0.008855903 |
| MPZL1 | 1.048451779 | 1.027590953 | 1.069736095 | 3.94E-06 |
| BACE1-AS | 1.20879181 | 1.094977082 | 1.334436732 | 0.000171068 |
| PES1 | 1.028733645 | 1.013173762 | 1.04453249 | 0.000269435 |
| CSTB | 1.006782557 | 1.002880732 | 1.010699563 | 0.00064509 |
| GLRX3 | 1.118757557 | 1.0599045 | 1.180878534 | 4.70E-05 |
| PPP1R14B | 1.007636774 | 1.001875602 | 1.013431076 | 0.009309332 |
| SYNGR2 | 1.009143148 | 1.001549128 | 1.016794748 | 0.018195877 |
| ZNF623 | 1.161050403 | 1.056274951 | 1.276218882 | 0.001971127 |
| THOC5 | 1.255384313 | 1.152017408 | 1.368026006 | 2.13E-07 |
| SGSM3 | 1.046479325 | 1.002570298 | 1.092311411 | 0.037770268 |
| PLEKHA8P1 | 1.864462881 | 1.482490883 | 2.344852083 | 1.00E-07 |
| ZNF485 | 1.795599614 | 1.423037757 | 2.265700932 | 8.08E-07 |
| PPP1R14BP3 | 1.008382529 | 1.000941851 | 1.015878518 | 0.027167883 |
| YWHAZ | 1.012466169 | 1.006635722 | 1.018330387 | 2.62E-05 |
| CNOT11 | 1.031709408 | 1.016786099 | 1.046851745 | 2.68E-05 |
| SAMD1 | 1.033745167 | 1.015452001 | 1.052367881 | 0.00026924 |
| CCDC137 | 1.04730652 | 1.022624055 | 1.072584731 | 0.000145584 |
| TMEM65 | 1.12305469 | 1.047407024 | 1.204165913 | 0.001107206 |
| SLC52A2 | 1.02663117 | 1.014074807 | 1.039343007 | 2.84E-05 |
| CEP164 | 1.43806632 | 1.164265629 | 1.7762568 | 0.000748075 |
| NCF2 | 1.042867811 | 1.02125491 | 1.064938108 | 8.55E-05 |
| POP1 | 1.384195827 | 1.205433387 | 1.589468243 | 4.06E-06 |
| IVD | 0.967325673 | 0.949388823 | 0.985601404 | 0.000503852 |
| MEX3A | 1.184272474 | 1.113609592 | 1.259419192 | 7.12E-08 |
| FAM89B | 1.049939654 | 1.01410707 | 1.08703835 | 0.005947611 |
| FHL3 | 1.067235605 | 1.035824593 | 1.099599144 | 1.96E-05 |
| AKAP8L | 1.068133367 | 1.015232944 | 1.123790257 | 0.010980699 |
| HMGA1 | 1.006259094 | 1.002934503 | 1.009594706 | 0.000219573 |

Table S3: M0RGs associated with the prognosis of patients with HCC in ICGC datasets using univariate Cox analysis.

| id | HR | HR.95L | HR.95H | pvalue |
| --- | --- | --- | --- | --- |
| SLC16A3 | 1.011904444 | 1.00364663 | 1.020230201 | 0.004645822 |
| LAPTM4B | 1.004601001 | 1.001483304 | 1.007728405 | 0.003796454 |
| TRAF4 | 1.035520956 | 1.008865604 | 1.062880572 | 0.008707132 |
| SCRIB | 1.032904603 | 1.009484047 | 1.056868528 | 0.005664261 |
| RCC2 | 1.022698588 | 1.002986859 | 1.042797711 | 0.023802155 |
| VPS37C | 1.122438124 | 1.022053437 | 1.232682457 | 0.015678975 |
| YWHAZ | 1.004618374 | 1.002161982 | 1.007080786 | 0.00022514 |
| C11orf80 | 1.080069482 | 1.009641968 | 1.155409663 | 0.02516354 |
| TONSL | 1.111209177 | 1.04057012 | 1.186643563 | 0.001651201 |
| EIF3D | 1.010722046 | 1.003021667 | 1.018481542 | 0.006272653 |
| ACTG1 | 1.000617517 | 1.000078415 | 1.00115691 | 0.024759292 |
| RRP1 | 1.053067029 | 1.011191149 | 1.096677091 | 0.012506948 |
| INTS8 | 1.060452753 | 1.006051625 | 1.117795561 | 0.028924717 |
| RTN3 | 1.028356768 | 1.014678367 | 1.042219562 | 4.26E-05 |
| SAMD1 | 1.060662355 | 1.005573629 | 1.118769029 | 0.0304479 |
| OTUB1 | 1.036341245 | 1.00341002 | 1.070353251 | 0.030266776 |
| P2RX4 | 1.065418174 | 1.029798887 | 1.102269481 | 0.000259738 |
| CCDC137 | 1.057664285 | 1.022221667 | 1.094335775 | 0.001265089 |
| MIF | 1.001489075 | 1.000415139 | 1.002564165 | 0.006564459 |
| NPM3 | 1.039216598 | 1.014418845 | 1.064620539 | 0.001797796 |
| RPL17 | 1.000485959 | 1.000161868 | 1.000810156 | 0.003291562 |
| OLA1 | 1.065857863 | 1.041757269 | 1.090516013 | 4.61E-08 |
| TAGLN2 | 1.0012074 | 1.000135246 | 1.002280703 | 0.027289542 |
| TMEM44 | 1.066785978 | 1.012491995 | 1.123991429 | 0.015275572 |
| LRRC37BP1 | 1.289441947 | 1.032989613 | 1.609561717 | 0.024650435 |
| SNRPD1 | 1.047237295 | 1.027131833 | 1.067736308 | 3.06E-06 |
| PES1 | 1.037256144 | 1.016387267 | 1.05855351 | 0.000419597 |
| EEF1E1 | 1.050921986 | 1.010528792 | 1.092929789 | 0.013001778 |
| YDJC | 1.047246254 | 1.008941133 | 1.087005654 | 0.015175458 |
| STIP1 | 1.025717902 | 1.01364711 | 1.037932437 | 2.62E-05 |
| MPZL1 | 1.036190788 | 1.008863268 | 1.06425854 | 0.00913216 |
| GLRX3 | 1.026298431 | 1.007324199 | 1.045630066 | 0.006402581 |
| PLEKHA8P1 | 1.396033018 | 1.077194445 | 1.809244558 | 0.011666279 |
| SYNGR2 | 1.010439005 | 1.005423305 | 1.015479727 | 4.31E-05 |
| PPP1R14B | 1.006939853 | 1.002342779 | 1.01155801 | 0.003053808 |
| HMGA1 | 1.003541889 | 1.00032097 | 1.006773179 | 0.031113104 |
| SNHG1 | 1.012698647 | 1.002854453 | 1.022639472 | 0.011345266 |
| E2F5 | 1.071423406 | 1.014262979 | 1.1318052 | 0.013653677 |
| ATIC | 1.050073838 | 1.034127262 | 1.066266315 | 3.90E-10 |
| ADSL | 1.042909217 | 1.024137663 | 1.062024836 | 5.80E-06 |


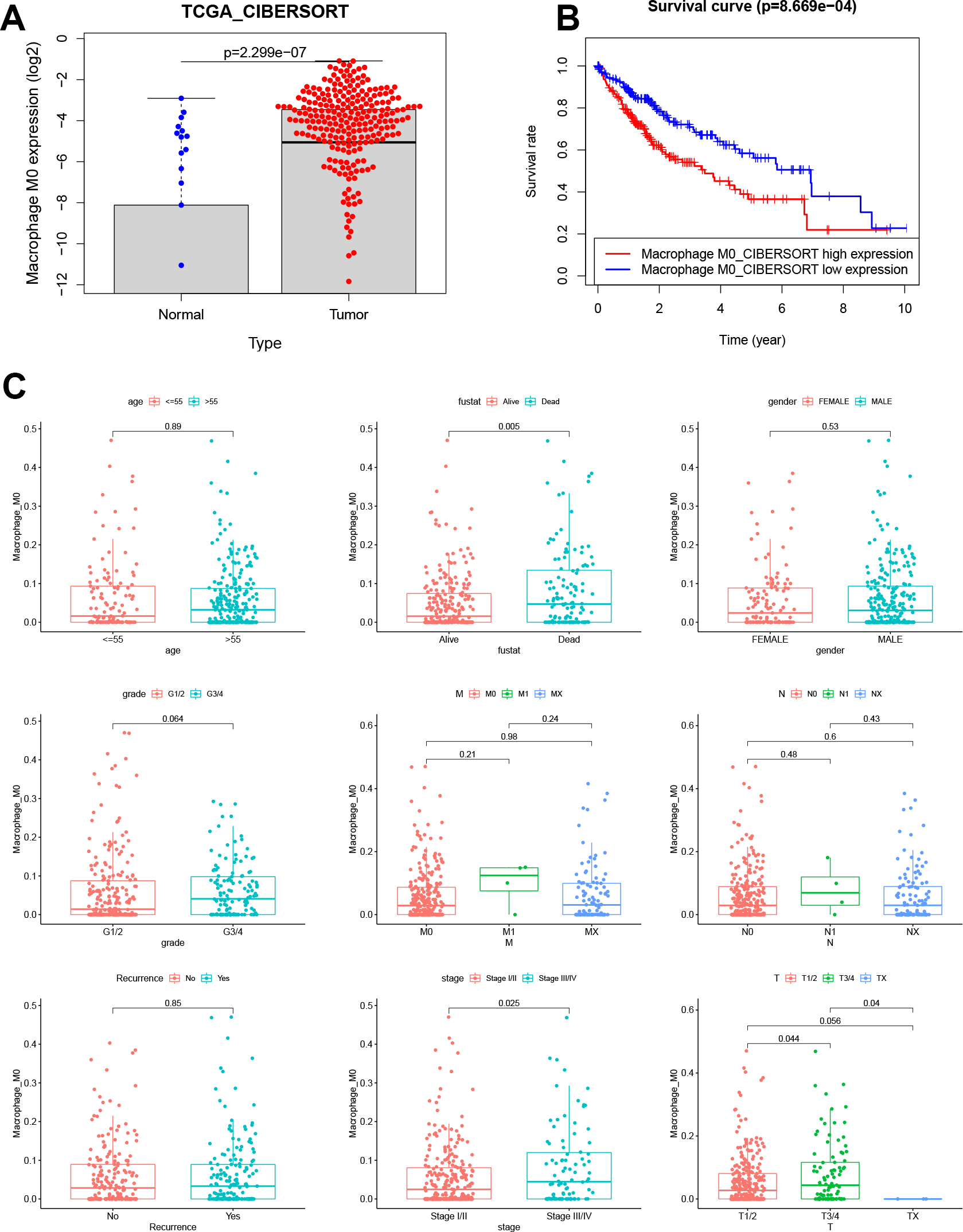


Figure S1. M0 macrophages in HCC.


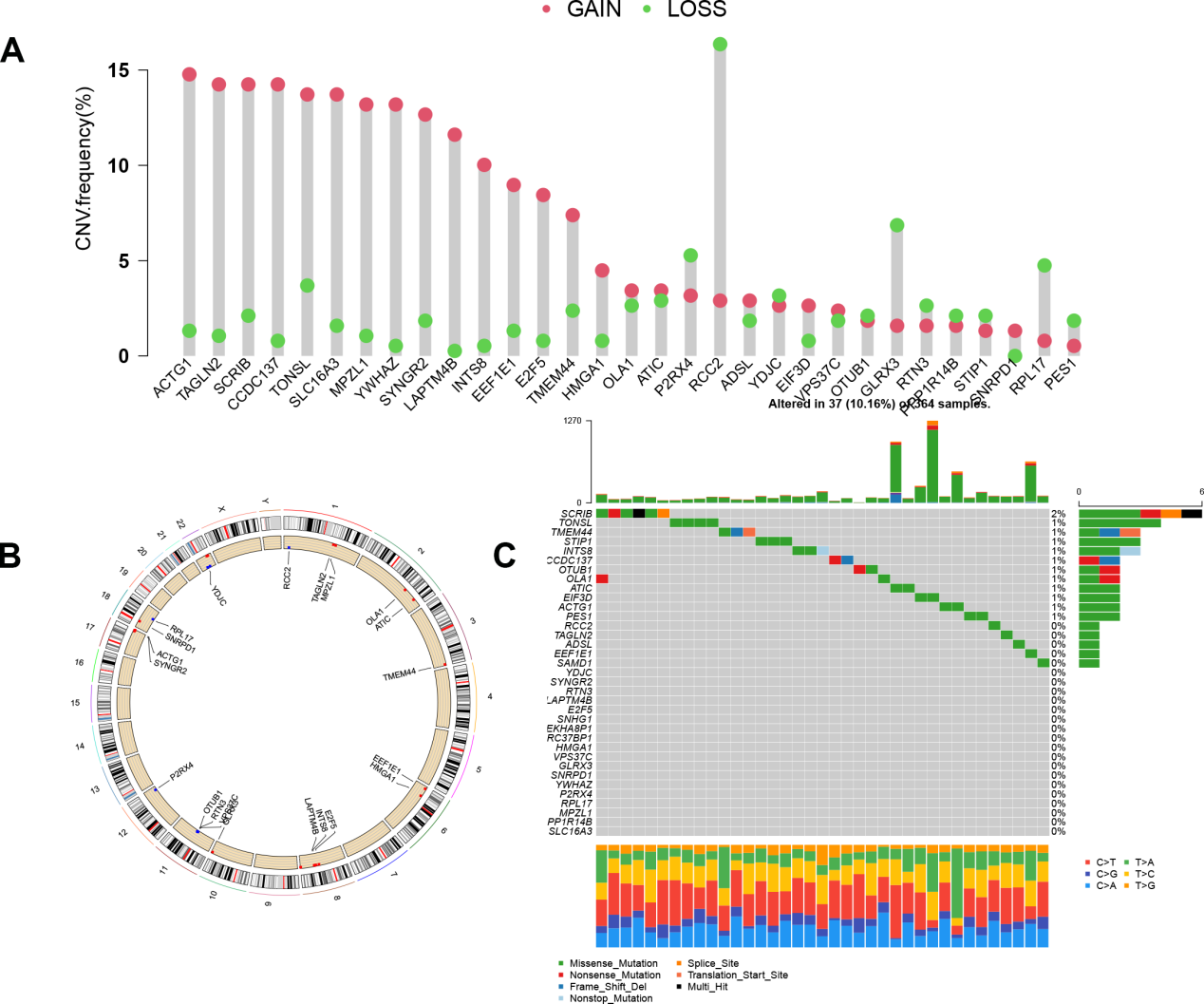


Figure S2. The CNV and mutation status of 35 M0RGs. A, The CNV status of 35 M0RGs. B, The mutation status of 35 M0RGs.


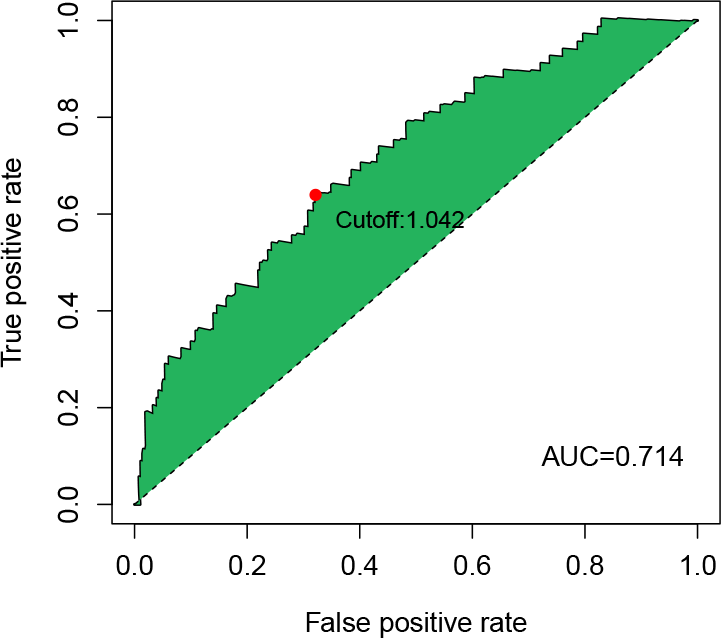


Figure S3. The best cutoﬀ value to distinguish the high-and low-risk groups.


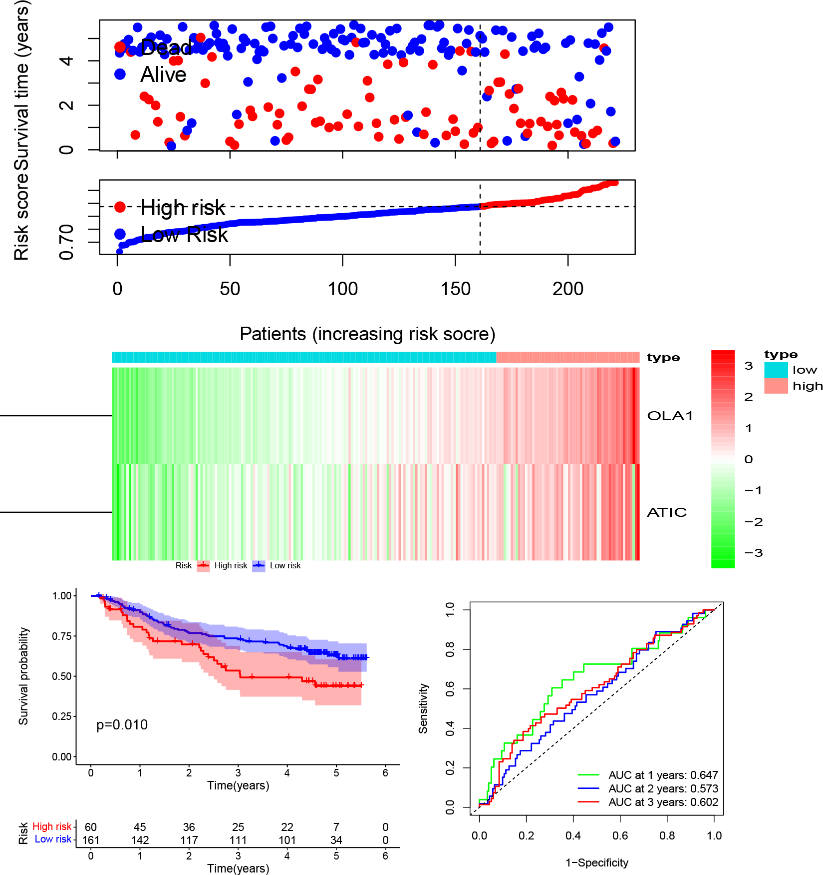


Figure S4 Prognostic model of the test (GSE14520) cohort. Risk score of the high and low groups. Heatmap of the expression of 2 M0RGs. Survival analysis of the high and low groups. The AUC of the ROC.

M0RGs: M0 macrophages-related genes; AUC: Area under curve; ROC: Receiver operating characteristic curve


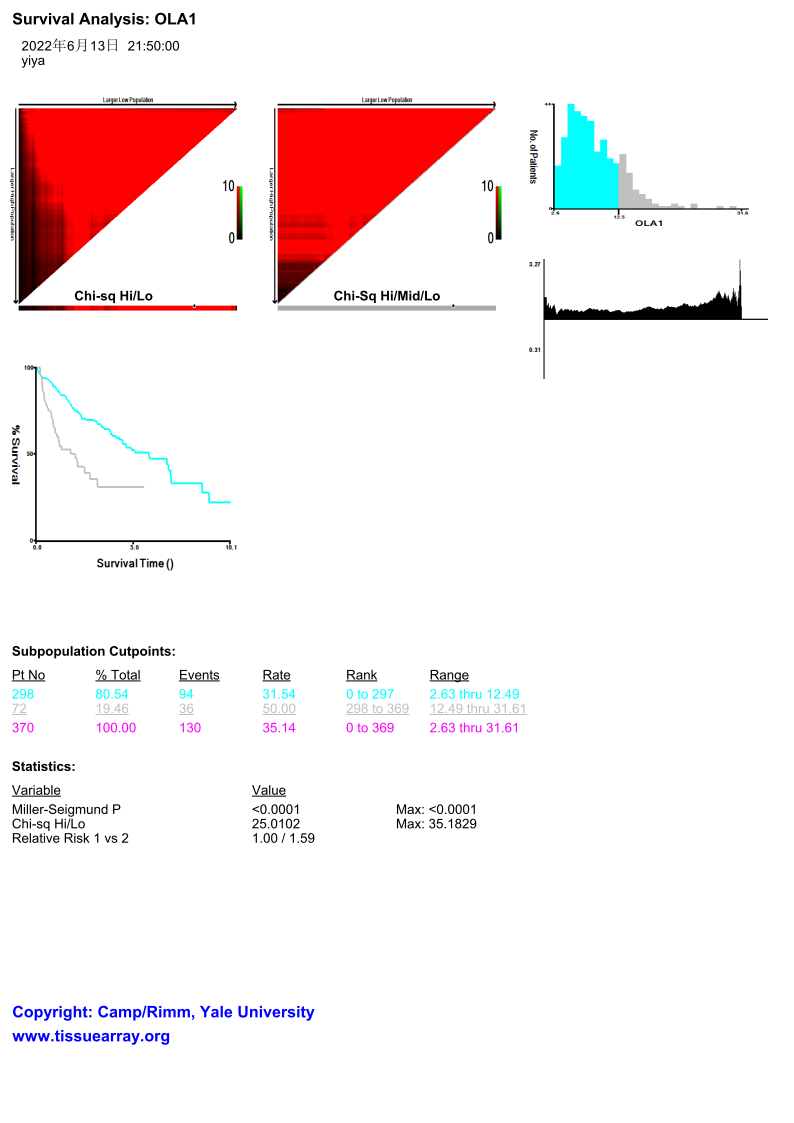


Figure S5 The survival analysis of OLA1 were analyzed using the X-Tile software


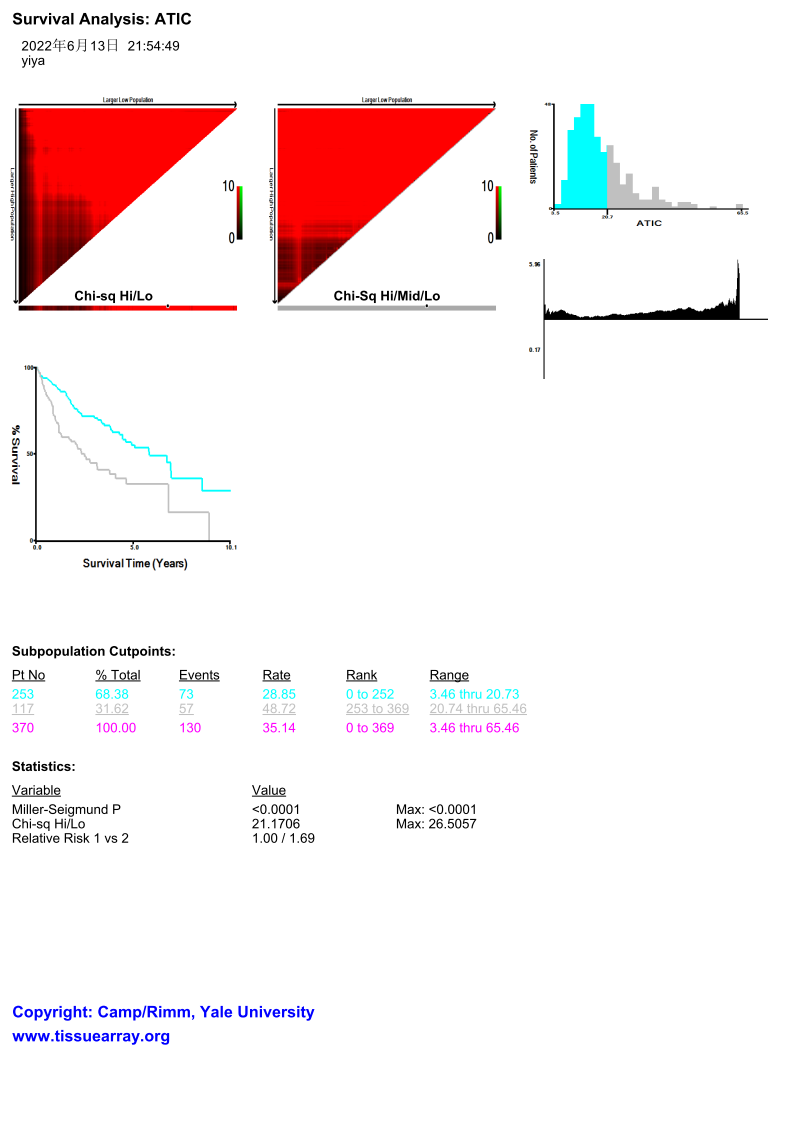


Figure S6 The survival analysis of ATIC were analyzed using the X-Tile software


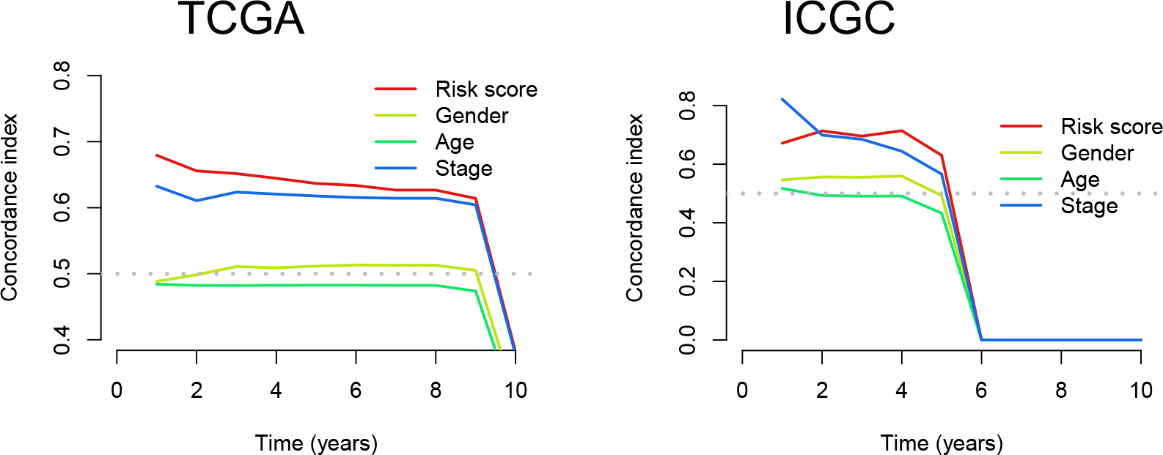


Figure S7. C-index for discrimination


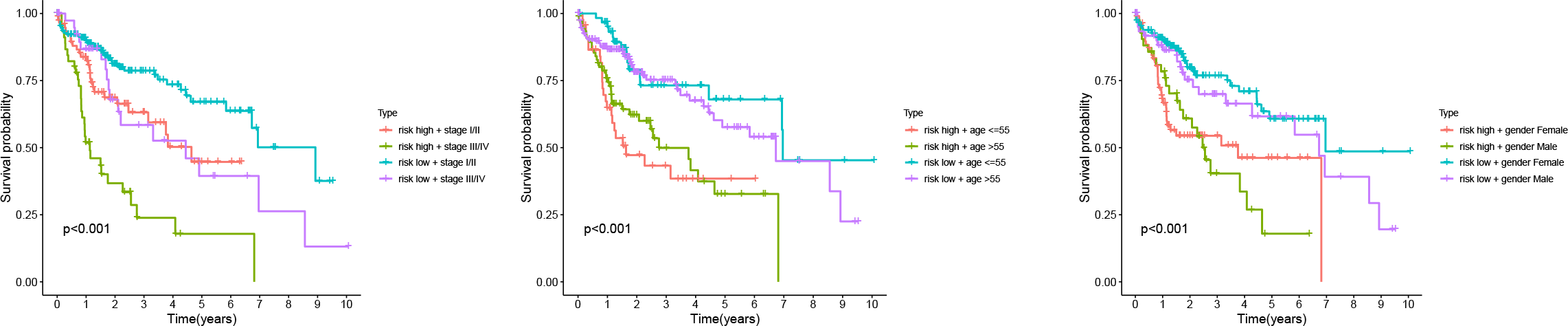


Figure S8. The HCC patients with high risk also had poor prognosis of OS with different clinical characters.


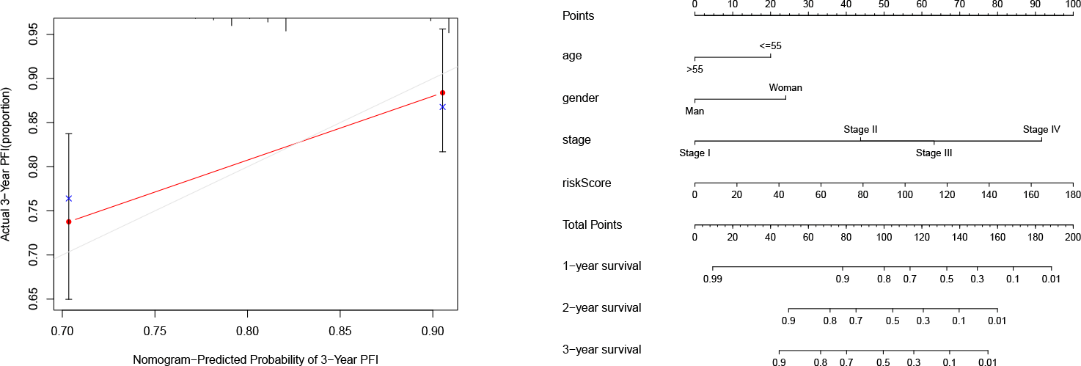


Figure S9. Construction of the nomogram in the ICGC dataset. The nomogram to predict the 3-year survival risk of HCC patients.


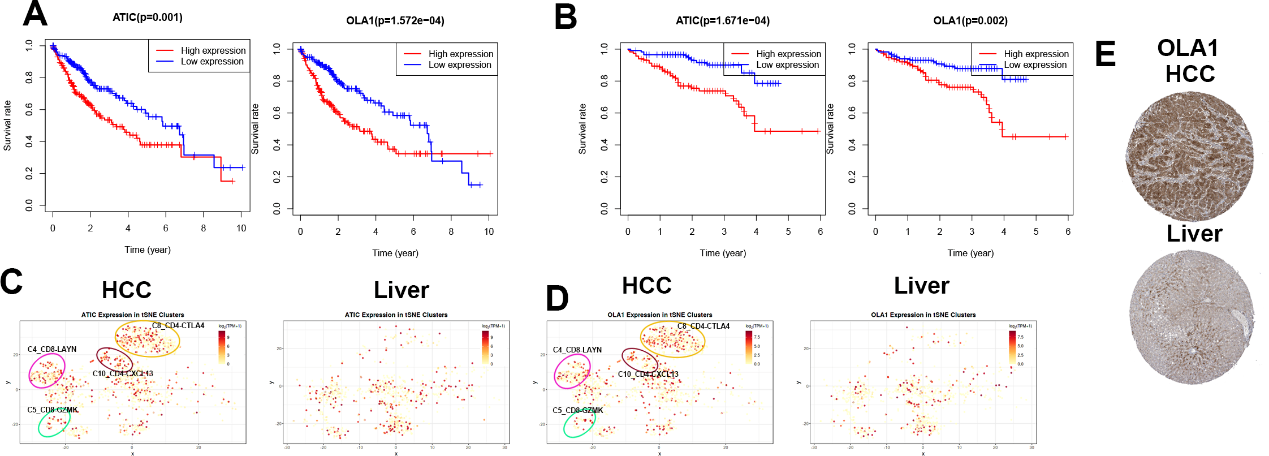


Figure S10．The expression and prognosis of M0RGs in HCC. A, The survival analysis of HCC with high/low ATIC and OLA1 in TCGA. B, The survival analysis of HCC with high/low ATIC and OLA1 in ICGC. C, ATIC and D, OLA1 expression in immune cells using tSNE cluster web tool. E, The protein levels of OLA1 in HCC using HPA dataset.


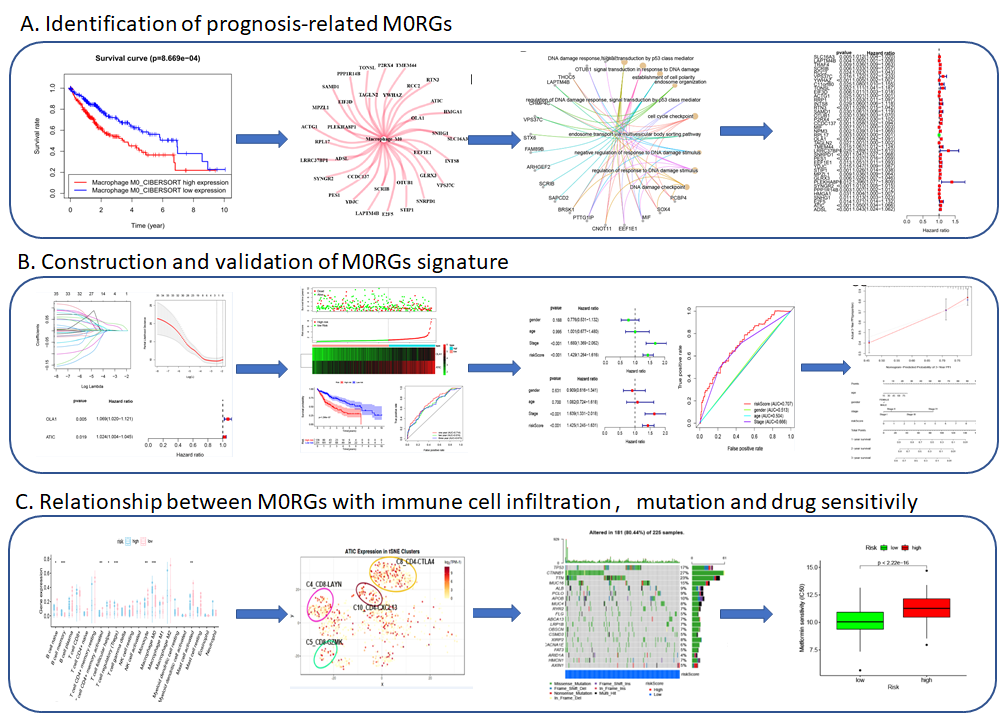


Figure S11 Schematic depicting the construction of an M0 macrophage-related prognostic model for hepatocellular carcinoma
